# CD32b defines distinct dendritic cell lineages generated from the culture of bone marrow with GM-CSF

**DOI:** 10.3389/fimmu.2026.1703978

**Published:** 2026-05-29

**Authors:** Wanho Choi, Seul Hye Ryu, Ji Soo Park, Da Eun Park, Min Kyung Chu, Hye Young Na, Chae Gyu Park

**Affiliations:** 1 Laboratory of Immunology, Severance Biomedical Science Institute, Yonsei University College of Medicine, Seoul, Republic of Korea; 2 Brain Korea 21 FOUR Project for Medical Science, Yonsei University College of Medicine, Seoul, Republic of Korea; 3 Department of Neurology, Severance Hospital, Yonsei University College of Medicine, Seoul, Republic of Korea; 4 Laboratory of Dendritic Cell Immunology, The Good Capital Institute for Immunology, Seoul, Republic of Korea

**Keywords:** CD32b, dendritic cells, GM-CSF, granulocyte-monocyte progenitor, *in vitro* differentiation, lineage heterogeneity, monocyte-dendritic cell progenitor, myeloid progenitors

## Abstract

Culturing bone marrow (BM) with granulocyte macrophage-colony stimulating factor (GM-CSF) is the most commonly used standard method of generating mouse dendritic cells (DCs) *in vitro*, although the development of those MHC II^hi^ GM-CSF–induced DCs (GM-DCs) remains poorly elucidated. Here, we have characterized that *in vitro*-generated GM-DCs from the BM comprise two distinct subsets distinguished by the expression of CD32b, arising at a different time from a separate progenitor lineage. Monocyte-dendritic cell progenitors (MDPs) give rise to CD32b^-^ GM-DCs within the first week of the BM culture, while granulocyte-monocyte progenitors (GMPs) generate CD32b^+^ GM-DCs at a slower rate and become numerically dominant in the later stages. In addition, there exist separate populations of pre-GM-DCs in the BM culture; Ly6C^-^CD32b^-^MHC II^int^ pre-GM-DCs for CD32b^-^ GM-DCs and Ly6C^-^CD32b^+^MHC II^int^ pre-GM-DCs for CD32b^+^ GM-DCs. These two GM-DC subsets also exhibit distinct functions; specifically, CD32b^+^ GM-DCs have enhanced capacity to stimulate CD4^+^ T cells. Notably, when adoptively transferred to mice treated with GM-CSF, GMPs selectively generate CD32b^+^ GM-DCs *in vivo* with delayed kinetics, compared to MDPs that give rise to heterogeneous GM-DC subsets *in vivo* rather quickly. Therefore, we have identified CD32b as a marker to differentiate two developmentally and functionally distinct GM-DCs, thus demonstrating the heterogeneity of GM-DCs, for the first time.

## Introduction

Dendritic cells (DCs) are professional antigen-presenting cells that bridge innate and adaptive immunity by initiating T cell responses to pathogens (1). DCs are broadly classified into plasmacytoid DCs (pDCs) and classical DCs (cDCs) based on their developmental origins and functions. pDCs, derived from common lymphoid progenitors (CLPs), are specialized for antiviral responses and characterized by their production of type I interferons (2). cDCs originate from myeloid progenitors and are further subdivided into type 1 (DC1) and type 2 (DC2) subsets. DC1s specialize in antigen cross-presentation to CD8^+^ T cells and express *IRF8*, *BATF3*, and *XCR1*, while DC2s activate CD4^+^ T cells better and display greater heterogeneity in surface markers and transcriptional programs (3).

Recent studies have highlighted the functional and developmental diversity within the DC2 compartment. Distinct DC2 subsets have been identified based on transcription factors such as RORγt (*RORC*) and T-bet (*TBX21*), as well as surface markers including *ESAM*, CD301b (*MGL2*), DC-SIGN (*CD209A*), and *CLEC12A* (4, 5). Also, this heterogeneity frequently overlaps with monocyte-derived cells including monocyte-derived DCs (MoDCs) (6).

Following the foundational discoveries that GM-CSF cultures of mouse BM or blood generate large numbers of DCs *in vitro* (7, 8), it was subsequently demonstrated that culturing human monocytes *in vitro* with GM-CSF and IL-4 yields MoDCs (9). Further research established that mouse monocytes generate MoDCs during inflammation *in vivo* (10, 11). Notably, studies utilizing GM-CSF and GM-CSF receptor knock-out mice have revealed distinct developmental requirements: some reports indicate that MoDCs can be generated *in vivo* independently of GM-CSF during inflammation (12), while others demonstrate that highly immunogenic, pathogenic MoDCs are strictly dependent on GM-CSF signaling (13). These parallel findings suggest the existence of multiple, context-dependent pathways for MoDC development, necessitating a more precise examination of their ontogenetic roots.

To investigate these distinct DC populations *in vitro*, two primary culture systems are conventionally employed: FLT3L-based cultures, which largely mirror the development of steady-state cDCs, and GM-CSF-based cultures, which are widely used to model inflammatory MoDCs (14). These *in vitro* platforms have become essential tools for deciphering the complex relationship between classical and inflammatory DC lineages.

Myeloid progenitors are classically divided into granulocyte-monocyte progenitors (GMPs) and monocyte-dendritic cell progenitors (MDPs), both of which give rise to monocytes, albeit with different differentiation potentials (15). MDPs are regarded as the main source of cDCs and macrophages, while GMPs primarily generate granulocytes and neutrophils (15, 16). However, emerging evidence suggests that GMP-derived monocytes may contribute to DC differentiation under specific conditions (14).

One such population, recently termed DC3s, is proposed to originate from Ly6C^+^ MDPs through a precursor stage known as pro-DC3s. DC3 subset is phenotypically defined by markers such as CD16/32 (FcγrIII/IIb; *FCGR3/FCGR2B*), CD172a (*SIRPA*), and *LYZ2* (4, 17) but its precise lineage and relationship to the other cDCs need further elucidation.

Culturing mouse bone marrow (BM) with granulocyte macrophage-colony stimulating factor (GM-CSF) has long been used as a standard *in vitro* model for generating CD11b^+^ DCs within approximately one week (18, 19). Historically, the conceptual framework for defining DC states has evolved in parallel with these culture models. In the original “immature/mature” model, CD11c^+^ cells in GM-CSF BM cultures were primarily classified into proliferating immature DCs and mature DCs based on their phenotype and function (8). However, this definition underwent a paradigm shift in the early 2000s with the introduction of the “steady-state versus infection” framework (20). This revised perspective redefined maturation as the functional transition of tolerogenic, steady-state DCs into immunogenic mature DCs specifically upon exposure to inflammatory stimuli. Since then, various nuanced models have emerged, such as the “immature/semi-mature/mature” model (21), “steady-state tolerogenic maturation” (22), and “homeostatic maturation” (23). These modern interpretations suggest that CD11c^+^ cells generated in the absence of exogenous inflammatory signals likely represent a steady-state immature stage, despite exhibiting a degree of phenotypic similarity to activated DCs.

Adding to these developments, Helft and colleagues proposed a classification dividing CD11c^+^ cells in GM-CSF BM cultures into MHC II^hi^ GM-DCs and MHC II^int^ GM-Macs based on functional capacity and transcriptomic profiling (24). Beyond these findings, numerous studies have since endeavored to interpret the heterogeneity of CD11c^+^ cells by distinguishing them into MHC II^hi^ and MHC II^lo/int^ subpopulations across various species and experimental contexts (25, 26). While MHC II^int^CD11c^+^ cells were thus characterized as a macrophage population, the heterogeneous nature of BM culture with GM-CSF has raised the possibility that GM-Mac may also contain precursors to GM-CSF-induced DCs (GM-DCs), which is a subject of ongoing debate (27, 28). Given the evolving nomenclature and conceptual ambiguity surrounding the developmental stages of *in vitro*-generated GM-DCs, there is a critical need to precisely characterize their function and developmental potential from an ontogenetic perspective.

In the present study, we evaluated the composition of GM-DCs in the BM culture and identified a developmentally and functionally distinct subset of CD32b^+^ GM-DCs that emerged slowly from GMPs. These CD32b^+^ GM-DCs exhibited delayed kinetics of appearance, unique pre-DC intermediates, and enhanced CD4^+^ T cell stimulating capacity, compared to early-arising CD32b^-^ GM-DCs which are derived from MDPs.

## Materials and methods

### Animals

C57BL/6 mice was purchased from Orient Bio (Seongnam, Republic of Korea), and C57BL/6-Tg(TcraTcrb)1100Mjb/J (OT-I), B6.Cg-Tg(TcraTcrb)425Cbn/J (OT-II), B6.SJL-Ptprc^a^Pepc^b^/BoyJ (CD45.1) mice were obtained from the Jackson Laboratory (Bar Harbor, ME). CD45.1^+^ OT-I and OT-II mice were bred in-house. All mice were maintained under specific pathogen-free (SPF) conditions at the Department of Laboratory Animal Resources of Yonsei University College of Medicine. Mice were used between 8–14 weeks of age, and both male and female animals were included. All experiments involving animals were performed according to the guidelines and protocols approved by the Institutional Animal Care and Use Committees (IACUCs) of Yonsei University College of Medicine (approval numbers: 2016-0040, 2017-0001, 2019-0024, 2019-0180, 2020-0003, 2021-0010, 2023-0162).

### Antibodies and reagents

Fluorochrome-conjugated monoclonal antibodies used in this study were purchased from BioLegend (San Diego, CA), BD Biosciences (San Jose, CA), Invitrogen (Carlsbad, CA), and eBioscience (San Diego, CA). Antibodies from BioLegend included anti-CD3 (17A2), CD4 (GK1.5), CD8α (53-6.7), CD11b (M1/70), CD11c (N418), CD14 (Sa14-2), CD16/32 (93), CD19 (6D5), CD24 (M1/69), CD25 (3C7), CD34 (RAM34), CD40 (FGK45), CD45.1 (A20), CD45.2 (104), B220/CD45R (RA3-6B2), TCRβ (H57-597), TCR Va2 (B20.1), CD80 (16-10A1), CD83 (Michel-19), CD86 (GL-1), CD103 (2E7), CD115 (AFS98), CD117 (2B8), CD127 (A7R34), FLT3 (A2F10), CD172a (P84), DEC205 (NLDC-145), CD206 (C068C2), CD301a (LOM-8.7), CD301b (URA-1), PD-L1 (10F.9G2), PD-L2 (TY25), Sca-1 (D7), H-2 MHC I (M1/42), I-A/I-E MHC II (M5/114.15.2), NK1.1 (PK136), CD49b (DX5), Ly6C (HK1.4), Ly6G (1A8), F4/80 (BM8), TER-119 (TER-119), 33D1 (33D1), CCR6 (29-2L17), CCR7 (4B12), CX3CR1 (SA011F11) and CLEC12A (5D3/CLEC12A). Additional antibodies from Invitrogen included anti-CD32b (AT130-2), anti-CD209a (MMD3) and anti-CD209b (22D1). Details of conjugated fluorochrome to each antibody are described in **Supplementary Table 1**. For transfection procedures, Lipofectamine™ 2000 reagent (Invitrogen) was used according to the manufacturer’s instructions. For cell proliferation assays, CellTrace™ CFSE (Thermo Fisher Scientific, Waltham, MA) was employed to label naïve T cells. Ovalbumin (Grade V) was obtained from Sigma-Aldrich (St. Louis, MO). Mouse GM-CSF was produced in-house as previously described (29). Briefly, culture supernatants from GM-CSF-producing CHO-S cells were harvested and purified using Anti-FLAG M1^®^ Agarose affinity gel (Merck Millipore, Burlington, MA). All cells were cultured in DMC7 (**DM**EM-based **C**TM supplemented with **7**% FBS) medium (29), which consisted of DMEM containing high glucose, L-glutamine, and sodium pyruvate (GE Healthcare Life Sciences, Pittsburgh, PA or Cytiva, Marlborough MA) supplemented with 7% fetal bovine serum (FBS, Cytiva), 1× non-essential amino acids, antibiotic-antimycotic, and Primocin™ (InvivoGen). The recipe of DMC7 is a modification of Kappler-Marrack’s DMEM-based CTM (complete tumor medium) (30) with reduced FBS content and without 2-mercaptoethanol.

### Primary cell culture

BM and spleen were harvested from mice after euthanasia by five minutes of CO_2_ inhalation (with filling 30% - 70% of cage per minute). According to the current guidelines for generation of murine BM-derived MoDC with GM-CSF (14), cells were cultured in 24-well tissue culture plates at densities of 1×10^6^cells/well (splenocytes) or 1×10^5^ cells/well (BM), and 48-well tissue culture plates at densities of 5×10^4^ cells/well (BM), depending on experimental design. The culture medium consisted of DMC7 medium supplemented with 1-5% (v/v) conditioned medium from GM-CSF-expressing cells, yielding a final GM-CSF concentration of approximately 0.1-0.5 μg/mL (14, 29). Half of the medium was replaced with fresh GM-CSF-containing DMC7 every 3–4 days. Following the culture period, non-adherent or loosely adherent cells were harvested exclusively via gentle pipetting to exclude adherent cells (i.e. macrophages). When performing co-culture experiments, CD45.1^+^ filler cells were seeded at 1×10^6^cells/well (splenocyte) or 5×10^4^ cells/well (BM), and CD45.2^+^ sorted cells were added at defined densities per condition. For the culture of progenitor subsets (GMPs, MDPs, etc.), FACS-sorted populations (CD32b^+^ GM-DCs, CD32b^+^ Pre-GM-DCs, etc.) from CD45.2 mice were seeded at defined densities (e.g., 5×10^2^ for GMPs, 1×10^4^ for CD32b^+^ GM-DCs, etc.) and co-cultured with CD45.1 whole BM filler cells (5×10^4^ cells/well) in GM-CSF-conditioned medium for 21 days.

### Tissue processing and single-cell suspensions

Tissues were harvested from mice after euthanasia by five minutes of CO_2_ inhalation (with filling 30% - 70% of cage per minute) or intraperitoneal injection of a mixture of 6.25 mg tiletamine and zolazepam (Zoletil^®^ 50, Virbac, Carros, France) and 0.58 mg xylazine hydrochloride (Rompun^®^, Bayer, Leverkusen, Germany) per mouse. For BM collection, femurs and tibias were isolated from the hind legs of mice, cleaned of muscle tissue, and disinfected in 70% ethanol. BM was flushed out using a syringe with 23-gauge needle, passed through 70–100 µm cell strainers, and pipetted to obtain single cell suspensions. The total number of BM cells was counted before or after RBC lysis using a hemocytometer. Erythrocytes were lysed using commercial RBC lysis buffer (eBioscience). For isolation of splenocyte and sdLN cells, spleens and sdLNs were mechanically disrupted using syringe plungers and filtered through nylon mesh (100 µm) (31). The total number of splenocytes and sdLNs was counted after RBC lysis and subjected to RBC lysis. For lung and liver digestion, mice were perfused with HBSS through the right ventricle. Lungs and livers were minced with razor blades and incubated with 1 mg/mL collagenase type IV or collagenase D at 37 °C for 40 minutes, followed by treatment with 20 mM EDTA for 5 minutes (31). Samples were filtered, lysed for RBCs, and washed thoroughly.

### Flow cytometry

Single-cell suspensions were incubated with anti-mouse Fc receptor blocking antibody (2.4G2) or fluorochrome-conjugated anti-CD16/32 antibody (93) for 20 minutes at 4 °C. Anti-CD32b (AT130-2) remains functional following Fc receptor blockade using 2.4G2 (32). Cells were then washed with FACS buffer consisting of DPBS supplemented with 2% FBS, 0.1% sodium azide, and 2 mM EDTA. Samples were stained with cocktails of fluorochrome- and/or biotin-conjugated monoclonal antibodies in 96-well V-bottom plates. Staining was performed for 30 minutes at 4 °C. For detection of intracellular molecules, surface staining was followed by fixation and permeabilization using commercial kits (e.g., Fixation Buffer/Permeabilization Wash Buffer, BioLegend) according to the manufacturer’s instructions. Dead cell discrimination was performed using Fixable Zombie Aqua™ dye (BioLegend), LIVE/DEAD™ Fixable Far Red viability dyes (Thermo Fisher Scientific). Antibody panels used for flow cytometry included lineage cocktails (e.g., CD3, CD11b, CD11c, CD19, B220, NK1.1, DX5, Ly6G, TER-119, MHC II, Sca-1, CD127), myeloid/DC markers (e.g., MHC II, CD11b, CD11c, CD32b, Ly6C), and precursor/progenitor markers (e.g., CD115, CD117, FLT3, CD34, Ly6C). Multiparameter flow cytometric analysis was performed on FACSVerse™ or BD LSRFortessa™ cytometers (BD Biosciences). Cell sorting was conducted using BD FACSAria™ II (BD Biosciences) at the Flow Cytometry Core Facility of Yonsei University College of Medicine. Collected data were analyzed using FlowJo software (BD Biosciences).

### Progenitor cell sorting

BM cells were harvested from CD45.2^+^ donor mice after euthanasia by five minutes of CO_2_ inhalation (with filling 30% - 70% of cage per minute) and prepared as single-cell suspensions by flushing femurs and tibias followed by RBC lysis. After blocking Fc receptors with fluorochrome-conjugated anti-CD16/32 antibody, cells were stained with a cocktail of fluorochrome-conjugated monoclonal antibodies against lineage markers (CD3, CD11b, CD11c, CD19, B220, NK1.1, DX5, Ly6G, TER-119, MHC II, Sca-1, CD127) and progenitor surface markers (CD115, CD117, FLT3, CD34, Ly6C). Lineage-negative cells were gated and further subdivided to define specific progenitor subsets. Monocyte-Dendritic cell Progenitor (MDP) was defined as Lin^-^CD117^+^CD115^+^CD16/32^-^CD34^+^Ly6C^-^FLT3^+^, Granulocyte-Monocyte Progenitor (GMP) as Lin^-^CD117^+^CD115^-^CD16/32^+^CD34^+^Ly6C^+^ FLT3^-^, and Monocyte Progenitor (MoP) as Lin^-^CD117^+^CD115^+^CD16/32^+^CD34^+^Ly6C^+^ FLT3^-^. These gating strategies were based on established definitions and adapted to suit each experimental condition (15). Cell sorting was performed on a BD FACSAria™ II cell sorter (BD Biosciences), and sorted cells were collected into FBS-coated tubes containing ice-cold DMC7 medium. The purity of sorted populations was routinely confirmed to be greater than 95% before use in downstream experiments.

### RNA extraction, sequencing and analysis

Total RNA was extracted from sorted DC populations (≥1×10^5^ cells) from BM, splenocytes, or cultured progenitor cells with the MiniBEST universal RNA extraction kit (TaKaRa Bio, San Jose, CA) or RNeasy Mini kit (Qiagen, Germantown, MD) according to the manufacturer’s instructions. RNA quality was assessed, and cDNA synthesis was performed using SMARTer Ultra Low Input RNA library kit (TaKaRa Bio). RNA sequencing (RNA-seq) procedure and analysis were performed by Macrogen (Seoul, Korea). RNA-seq libraries were sequenced on Illumina NovaSeq (Illumina, San Diego, CA). Raw reads were preprocessed to remove low-quality and adapter sequences. Reads were aligned to the *Mus musculus* reference genome (*mm10*) using HISAT2 v2.1.0 or STAR aligner. Transcript quantification was performed using StringTie v1.3.4d. Multidimensional scaling (MDS) was used to visualize sample similarity using Euclidean distance. Hierarchical clustering was performed with complete linkage, and differential expression analysis applied |fold change| ≥ 2 threshold. Visualization was performed with GraphPad Prism. Venn diagrams were generated using InteractiVenn, and gene set enrichment analysis (GSEA) was conducted with MSigDB gene sets.

### ELISA

BM cells were seeded at 1×10^5^ cells per well in 24-well plates and cultured for 21 days with GM-CSF–conditioned medium, which was replaced twice weekly. At each week, cells from one plate were harvested, and the culture supernatants were collected and filtered. The undiluted supernatant from each time point was used as a representative sample for ELISA. Mouse TNF-α levels were quantified using 100 μL of supernatant according to the manufacturer’s instructions (BioLegend). Two biologically independent experiments were performed using pooled BM cells from two mice per experiment.

### Antigen presentation and T cell proliferation assays

For *in vitro* antigen presentation assays, cultured cells were pulsed with 100 µg/mL of OVA (Grade V) (Sigma-Aldrich, Saint Louis, MO) at 37 °C for 30 minutes to 1 hour. Cells were then harvested, washed thoroughly, and surface-stained to sort APCs (e.g., MHC II^hi^CD11c^+^, CD32b^+/-^) using FACSAria™ II. Naive OT-I (CD8^+^) and OT-II (CD4^+^) T cells were enriched from spleens of transgenic mice using magnetic depletion methods with biotinylated antibodies against CD19, CD11b, NK1.1, CD25, CD44, MHC II, and CD4/CD8 (as appropriate), followed by Dynabeads™ Biotin Binder (Thermo Fisher Scientific). Purified T cells were labeled with 5 µM CFSE at 37 °C for 10 minutes and washed before use. T cells (5×10^4^/well) were co-cultured with graded doses of sorted APCs in 96-well round-bottom tissue culture plates with 57.2mM 2-mercaptoethanol contained DMC7 medium. T cell proliferation was assessed by CFSE dilution after 3–4 days using flow cytometry. Pulsed pre-DCs and T cells only were used as controls.

### Adoptive cell transfer

Adoptive transfer experiments were performed using sorted progenitor cells (e.g.,GMP, MDP, etc.) as donor populations. BM cells were harvested from CD45.2^+^ donor mice and stained for surface markers to isolate defined progenitor subsets via flow cytometric sorter. A total of 1×10^4^ - 4×10^4^ CD45.2^+^ sorted progenitor cells were transferred intravenously via tail vein into CD45.1^+^ recipient mice. Recipient mice were treated with 10 µg/100 µL of GM-CSF (produced in-house) via subcutaneous injection every other day for 4 or 7 days. GM-CSF treatment was initiated immediately after adoptive transfer. Transferred cells were tracked in recipient BM, spleens, sdLNs, and livers using CD45.1/CD45.2 congenic markers. Phenotypic analysis, including the expression of MHC II and CD32b, was performed by multicolor flow cytometry at designated time points after transfer.

### Statistical analysis

Data are presented as mean ± SD or median with interquartile range (IQR), as appropriate. For normally distributed data (mean ± SD), comparisons between groups were performed using two-way ANOVA. For non-parametrically distributed data (median with IQR), statistical significance was determined by multiple Mann-Whitney U tests, with p-values adjusted using the Bonferroni correction for multiple comparisons. Statistical significance was defined as *p* ≤ 0.05 (*), p ≤ 0.01 (**), p ≤ 0.001 (***), p ≤ 0.0001 (****). All graphs were generated and analyzed using GraphPad Prism 10.

### Data availability

All the RNA-seq data generated during our study have been deposited and are available at the Gene Expression Omnibus (GEO) database under the accession numbers GSE304117. All other data and materials supporting the findings in this report are available from the first author upon request. Source data are provided with this paper.

## Results

### DCs are generated primarily from GMPs in the culture of splenocytes with GM-CSF

In our previous study (33), we observed the *in vitro* generation of MHC II^hi^ CD11c^+^ CD11b^+^ DCs in the extended, i.e., more than 2-week culture of splenocytes with GM-CSF. Most splenocytes diminished within the first week of the GM-CSF culture, followed by a gradual increase in the number of CD11b^+^ cells, peaking at the third week, and then declining. Among those newly generated CD11b^+^ cells, MHC II^hi^ GM-DCs also increased over time, reaching a peak at the third week of the culture of splenocytes (**Supplementary Figures 1A**, **S17A**) where a lineage-negative (Lin^-^) CD117^+^ myeloid progenitor population was identified to differentiate into MHC II^hi^ CD11c^+^CD11b^+^ GM-DCs in the presence of GM-CSF (33). To generate DCs, non-adherent cells were harvested from cell cultures supplemented with 1%–5% GM-CSF supernatant (approx. 100–500 ng/ml). This concentration range aligns with current guidelines, which recommend 400–800 U/ml (approx. 80–160 ng/ml) for robust DC generation (14). Furthermore, as we previously demonstrated (29), while total yield may vary, the kinetics and phenotypic frequency of DC development remain essentially identical at any GM-CSF concentration above 0.1% (10 ng/ml). Therefore, the culture conditions employed in this study provide a reliable and standard-compliant environment for DC differentiation.

**Figure 1:**
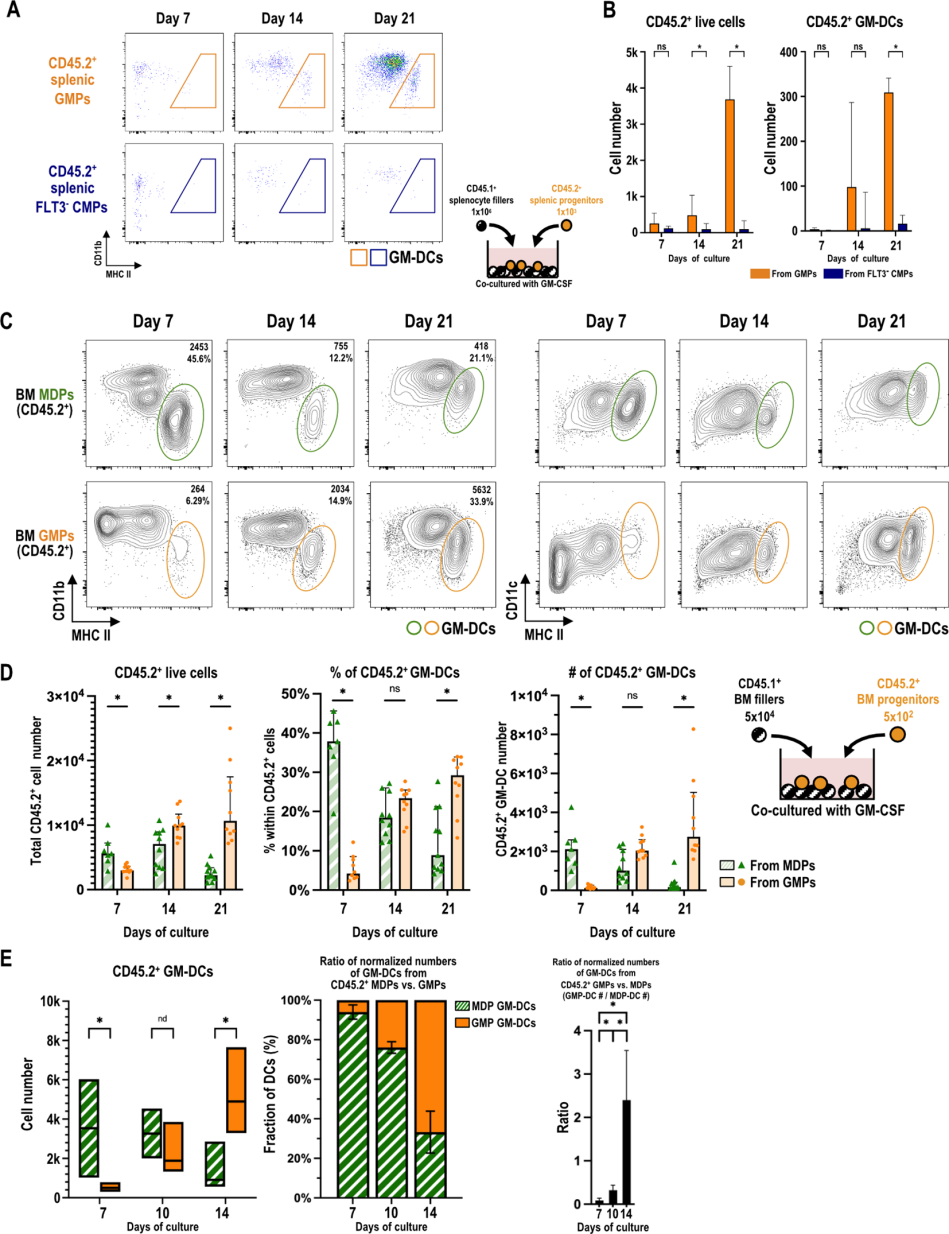
GMPs generate DCs with delayed kinetics compared to MDPs in GM-CSF culture. **(A, B)** Representative flow cytogram and **(B)** cell number of co-culture of sorted CD45.2^+^ splenic progenitors (splenic GMPs and FLT3^-^ CMPs) and CD45.1^+^ filler splenocytes with 1% GM-CSF-conditioned medium. CD45.1^+^ filler splenocytes were seeded at a density of 1×10^6^ cells and CD45.2^+^ sorted splenic progenitor cells were seeded at a density of 1×10^3^ cells in 24-well plate. Data are shown from two independent experiments in triplicate. **(C, D)** Representative flow cytogram, **(D)** cell number and proportion of culture of sorted BM GMPs and BM MDPs with GM-CSF. CD45.1^+^ BM fillers were seeded at a density of 5×10^4^ cells and sorted CD45.2^+^ BM progenitor cells were seeded at a density of 5×10^2^ cells in 48-well plate. Each dot represents one well of cultured cells. Data were pooled from three independent experiments, with variable replicate numbers per condition (n = 7–11). **(E)** Cell number and proportion of dendritic cells from co-culture of CD45.2^+^ BM GMPs and MDPs with CD45.1^+^ BM fillers and their reciprocal progenitor counterparts under 3% GM-CSF-conditioned medium. CD45.2^+^ and CD45.1^+^ progenitors were co-seeded at a density of 5×10^2^ cells, together with 5×10^4^ CD45.1^+^ filler cells, in 24-well plates. Data are shown from three independent experiments, with n = 2, 3, and 3 samples, respectively. Statistical significance was determined by multiple Mann-Whitney U tests, and p-values were adjusted using the Bonferroni correction for multiple comparisons. Error bars indicate median with interquartile range (IQR). ns, not significant; *p ≤ 0.05.

To further investigate the developmental origin of those GM-DCs in the splenocyte culture, we applied the classification scheme of BM myeloid progenitors (15, 34, 35) (**Supplementary Figure 2A**) to splenic progenitor populations (**Supplementary Figure 2B**). Both BM and spleen contained approximately 10^8^ total cells, but the BM had ten times more myeloid progenitors than the spleen (**Supplementary Figure 1B**). Besides, among the myeloid progenitor subsets, specifically GMPs and FLT3-negative common myeloid progenitors (FLT3^-^ CMPs) were consistently detected at significant levels in the spleen (**Supplementary Figure 2B**). Thus, we tested the differentiation potential of the two myeloid progenitors in the spleen, i.e., GMPs and FLT3^-^ CMPs. Respective progenitor cells were isolated from the spleen of CD45.2^+^ mice and co-cultured with the filler splenocytes from congenic CD45.1^+^ mice in the presence of GM-CSF (**Supplementary Figure 3**). MHC II^hi^ CD11c^+^ CD11b^+^ GM-DCs effectively developed from the culture of splenic GMPs, but not from that of splenic FLT3^-^ CMPs (**Figures 1A, B**; **Supplementary Figures 3B**, **S17B, C**). Therefore, splenic GMPs are the primary source of MHCII^hi^ CD11c^+^ CD11b^+^ GM-DCs in the culture of splenocytes.

### MDPs and GMPs exhibit distinct kinetics of DC development in the culture of BM with GM-CSF

We set out to investigate the role and contribution of GMPs to GM-DC production in the culture of BM with GM-CSF, where MDPs had been recognized as the primary source of GM-DCs (14–16, 24). CD45.2^+^ MDPs and GMPs were respectively isolated from the BM (**Supplementary Figure 4A**) and co-cultured with CD45.1^+^ BM filler cells in the presence of GM-CSF (**Supplementary Figures 4B****;** **S17D**). Both BM progenitors in the culture gave rise to MHC II^hi^ GM-DCs. While MDP-derived GM-DCs (MDP GM-DCs) reached their highest numbers by the first week, GMP-derived GM-DCs (GMP GM-DCs) exhibited delayed kinetics with their numbers peaking after the second week of GM-CSF culture (**Figures 1C, D**).

Then, we conducted reciprocal co-culture experiments to determine if the varying rates of GM-DC generation stemmed from the intrinsic cellular characteristics of progenitors, as opposed to influences from the excess or lack of competing progenitors in the culture with BM fillers. To ensure that the differential numbers of competing progenitors in the culture did not influence the outcome, we isolated equal numbers of MDPs and GMPs reciprocally from the BM of CD45.1^+^ and CD45.2^+^ mice, then co-cultured them with BM fillers (**Supplementary Figures 4C**, **S17E**). Consistent with the aforementioned results, reciprocal co-culture experiments replicated the differential development kinetics, with MDP GM-DCs predominating at early time points and GMP GM-DCs becoming the major population in the later stages of culture. (**Figure 1E**; **Supplementary Figures 4C**, **S17E**). Therefore, the distinct kinetics of GM-DC development from MDPs versus GMPs in the BM culture with GM-CSF seem to be intrinsic properties of the respective progenitors.

### BM myeloid progenitors differentiate into GM-DCs with two distinct kinetic patterns

**Figure 2:**
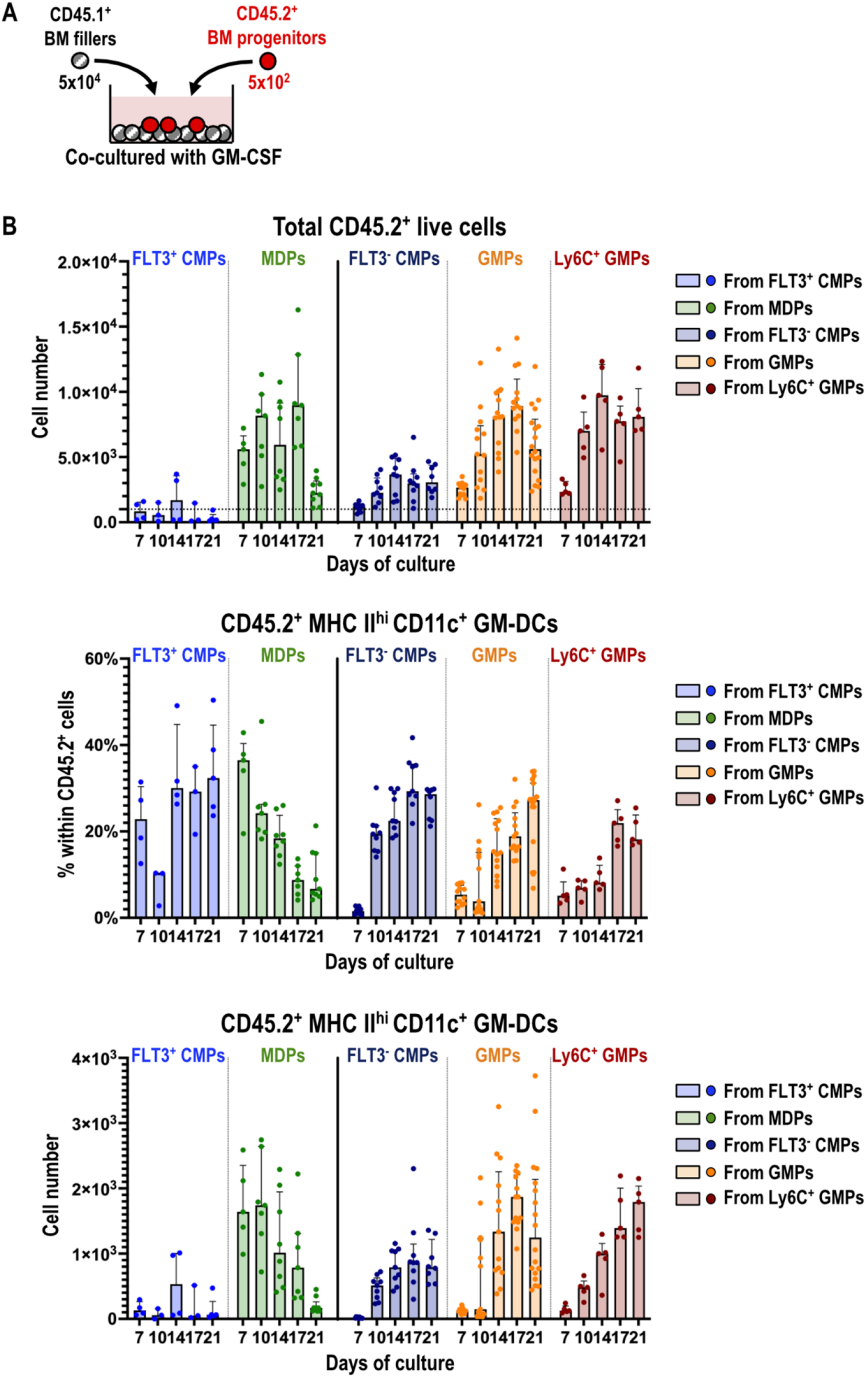
Distinct progenitor subsets follow different GM-DC differentiation kinetics from culture with BM fillers. **(A)** Scheme of the co-culture system with 3% GM-CSF-conditioned medium. CD45.1^+^ BM filler cells were seeded at a density of 5×10^4^ cells and sorted CD45.2^+^ progenitor cells were seeded at a density of 5×10^2^ cells in 48-well plate. **(B)** Time-course analysis of GM-DC differentiation from various BM myeloid progenitors cultured with BM filler cells for 21 days. DCs were quantified as MHC II^hi^CD11c^+^ cells. Each dot represents one well of cultured cells. Data were pooled from three independent experiments, with variable replicate numbers per condition (n = 5–12). Error bars indicate median with interquartile range (IQR).

Having confirmed that both GMPs and MDPs can generate GM-DCs in the culture of BM with GM-CSF, we proceeded to evaluate and compare the GM-DC differentiation potential of other myeloid progenitor subsets within the BM. The respective myeloid progenitors were isolated from the BM of CD45.2^+^ mice and co-cultured with CD45.1^+^ BM filler cells and GM-CSF (**Figure 2A**). All myeloid progenitor subsets, except for FLT3^+^ CMPs, generated more than double the quantity of GM-DCs relative to their initial input during the culture period (**Figure 2B**). As above, we observed the rapid generation of GM-DCs, with their numbers peaking in a week, only from the culture of MDPs. In contrast, both cultures of FLT3^-^ CMPs and Ly6C^+^ GMPs displayed a delayed GM-DC differentiation, consistent with the pattern observed in GMPs (**Figure 2B**). In addition, when MDPs, GMPs, and Ly6C^+^ GMPs were cultured respectively with GM-CSF in the absence of BM filler cells, we still observed two distinct patterns of GM-DC differentiation kinetics (**Supplementary Figures 5A, B****;** **S17F**). Similarly, doubling the initial input of both these progenitors and the BM filler cells did not alter these kinetic profiles, with the two distinct patterns remaining clearly observable (**Supplementary Figures 5C**, **S17G**). Our findings thus indicate that GM-CSF-responsive progenitors in the BM take one of two distinct developmental trajectories to generate GM-DCs during the culture, as evidenced by their binary pattern of differentiation kinetics.

**Figure 3:**
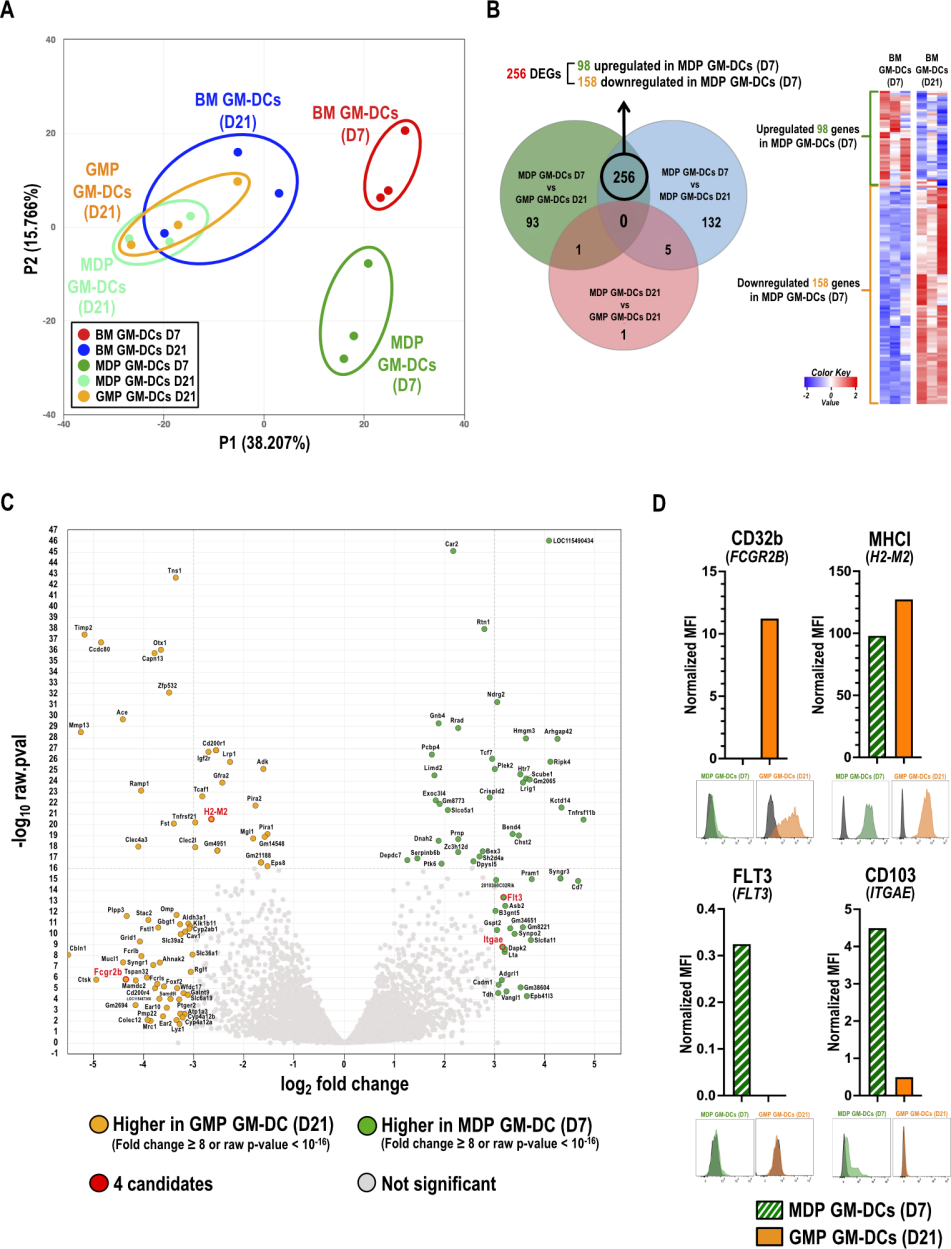
CD32b distinguishes transcriptionally and functionally distinct GM-DC subsets derived from MDPs and GMPs. **(A)** Principal component analysis (PCA) of bulk RNA-seq data from GM-DCs sorted on day 7 and day 21. DCs were generated from cultures of whole bone marrow (BM), sorted MDPs, or sorted GMPs with GM-CSF. **(B)** Heatmap showing the expression of genes upregulated and downregulated in MDP GM-DCs at day 7, identified through Venn diagram analysis and mapped onto BM GM-DCs at both time points. **(C)** Volcano plot of differentially expressed genes (DEGs) between MDP GM-DCs (day 7) and GMP GM-DCs (day 21). The thresholds for significance (dotted lines) are set at a log_2_(fold change) ≥ 3 or raw *p*-value ≤ 10^-16^. **(D)** Flow cytometric validation of selected surface marker candidates identified in the volcano plot **(C)**. Normalized Mean fluorescence intensity (MFI) of four representative markers is shown for each DC subset. Normalized MFI was calculated by subtracting the isotype control mean fluorescence from the sample mean fluorescence, and then dividing the result by the isotype control mean. Representative histograms are shown below the bar graphs, with the isotype control overlaid in gray. Data are representative of two independent experiments.

We conducted short-term (one and three-day) cultures of FLT3^+^ CMPs, FLT3^-^ CMPs, MDPs, and GMPs with GM-CSF (without BM filler cells) to characterize their potential for differentiating into downstream progenitors, monitoring their progressive differentiation (**Supplementary Figure 6A**). FLT3^+^ CMPs primarily gave rise to MDPs and CDPs by culture day 1, with subsequent differentiation into monocyte progenitors (MoPs) by culture day 3 (**Supplementary Figure 6B**). Likewise, MDPs initially differentiated into CDPs and MoPs by culture day 1, and then went on to form MoPs by culture day 3 (**Supplementary Figure 6C**). In stark contrast, FLT3^-^ CMPs and GMPs in culture were devoid of MDPs and CDPs, indicating a direct pathway to MoPs via Ly6C^+^ GMPs (**Supplementary Figures 6D, E**). The convergence of both lineages at the MoP stage is consistent with our RNA-seq analysis, which showed that MDP- and GMP-derived GM-DCs at day 21 exhibit nearly identical transcriptomic profiles (**Figures 3A, B**). Taken together, GM-DCs in BM culture appear to arise through two distinct trajectories: an early pathway from MDP-CDP lineages and a later one from GMP(MDP)-MoP lineages.

### FLT3 and CD32b distinguish GM-DCs derived from MDP versus GMP progenitor lineages

Our next step was to identify molecular markers or signature genes that can distinguish between the two kinetically unique pathways of cellular differentiation into GM-DCs within BM culture. Bulk RNA sequencing (RNA-seq) was performed on MHC II^hi^ GM-DCs obtained from five distinct culture conditions: BM cultures at day 7 and day 21, MDP cultures at day 7 and day 21, and GMP cultures at day 21. Principal component analysis (PCA) clearly separated the transcriptomes of BM-derived GM-DCs (BM GM-DCs) from day 7 and day 21 of culture. The moderate separation of day 7 transcriptomes from BM GM-DCs and MDP GM-DCs pointed to heterogeneity in the early-stage GM-DC population. In contrast, the day 21 transcriptomes from BM GM-DCs, GMP GM-DCs, and MDP GM-DCs all clustered together, indicating that prolonged culture with GM-CSF led to a convergence of gene expression regardless of their original progenitor lineage (**Figure 3A**).

Next, we analyzed the differentially expressed genes (DEGs) by comparing MDP GM-DCs at day 7, MDP GM-DCs at day 21, and GMP GM-DCs at day 21. The analysis identified 256 DEGs that distinguished day 7 MDP GM-DCs from both day 21 MDP GM-DCs and day 21 GMP GM-DCs (**Figure 3B**, left Venn diagram). As expected, very few DEGs were observed between MDP GM-DCs at day 21 and GMP GM-DCs at day 21. Notably, the DEGs upregulated in day 7 MDP GM-DCs showed similarly higher expression in day 7 BM GM-DCs, while the DEGs downregulated in day 7 MDP GM-DCs exhibited higher expression in day 21 BM GM-DCs (**Figure 3B**, right panel). Therefore, the expression patterns of DEGs suggest a temporal shift in the dominant progenitors for GM-DCs in BM culture.

We next sought to define cell-surface markers that distinguish the two kinetically distinct GM-DC populations in BM culture: Day 7 MDP GM-DCs and Day 21 GMP GM-DCs. A transcriptomic comparison of these two populations yielded a list of candidate DEGs, from which we selected four DC-related, surface-expressed genes (**Figure 3C**). As shown in **Figure 3D**, flow cytometric analysis suggested that FLT3 and CD32b could potentially distinguish the two GM-DC populations. While our RNA-seq data and normalized Mean Fluorescence Intensity (MFI) indicated that FLT3 might differentiate these populations, its actual surface expression on MDP-derived GM-DCs is too weak to allow for practical separation via flow cytometric sorting. Therefore, while FLT3 serves as a distinct “transcriptomic signature” for MDP GM-DCs, it is not a viable surface marker for physical isolation. In contrast, CD32b was expressed robustly and exclusively on Day 21 GMP-derived GM-DCs, whereas other tested markers were less effective in distinguishing the two populations; for instance, MHC class I was expressed at similar levels, while the difference in CD103 expression was insufficient to clearly separate them (**Figure 3D**). The selective expression of FLT3 and CD32b appears to define the two distinct progenitor lineages of GM-DCs. In the BM, one lineage (FLT3^+^ CMPs-MDPs-CDPs) expresses FLT3 but not CD32b, while the other (FLT3^-^ CMPs-GMPs-MoPs) expresses CD32b but not FLT3 (**Supplementary Figure 2A** (15)). Therefore, it is likely that the day 7 FLT3^+^ MDP GM-DCs and the day 21 CD32b^+^ GMP GM-DCs differentiate via these respective developmental trajectories.

### CD32b expression reflects the progenitor origin of GM-DCs in BM culture

Although FLT3 and CD32b were specific markers for MDP GM-DCs and GMP GM-DCs respectively, they exhibited markedly different signal intensities by flow cytometry. Anti-CD32b provided a strong signal, while the anti-FLT3 signal was only marginally positive above its isotype control (**Figure 3D**). Therefore, only CD32b expression could reliably distinguish the day 7 BM GM-DCs, which are presumed to be MDP-derived, from the day 21 BM and splenocyte-derived (Sp) GM-DCs, both of which are expected to be mainly GMP-derived (**Supplementary Figures 7A, B**). Specifically, we further examined CD16 (*FCGR3*), which is frequently co-analyzed with CD32b (*FCGR2B*). Both the DEG levels and surface expression of CD16 in D21 GMP-derived GM-DCs were significantly lower than those of CD32b (**Supplementary Figures 7C, D**). These results indicate that while anti-CD16/32 staining is commonly utilized, it is specifically CD32b, rather than CD16, that should be targeted for accurate discrimination.

**Figure 4:**
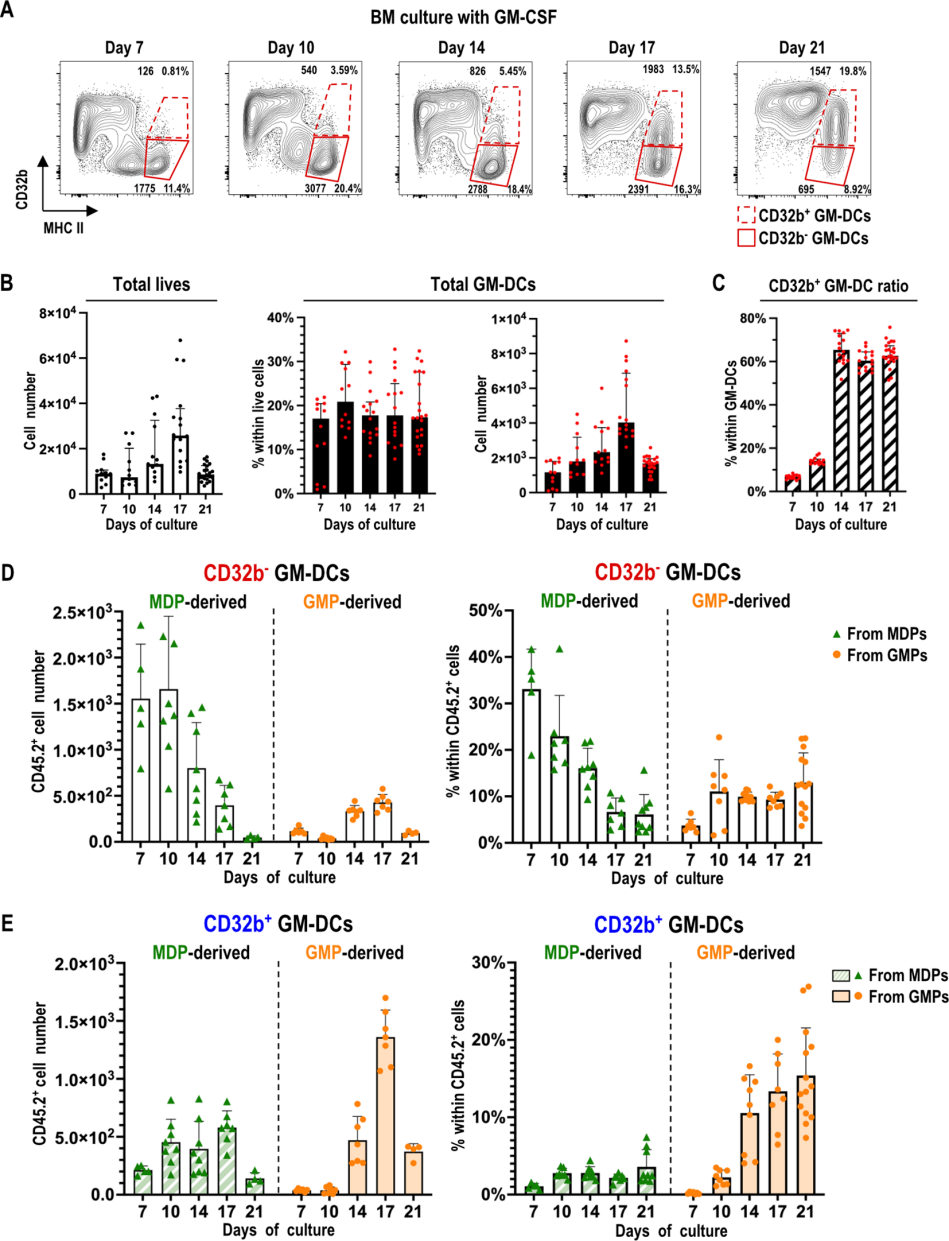
CD32b distinguishes GMP-derived GM-DCs that emerge with delayed kinetics in BM culture with GM-CSF. **(A)** Representative flow cytogram of MHC II and CD32b expression in BM cells cultured with 3% GM-CSF-conditioned medium for a 21 days. BM cells were seeded at a density of 5×10^4^ cells in 48-well plate. MHC II^hi^ GM-DCs were subdivided into CD32b^-^ GM-DCs (red solid lines) and CD32b^+^ GM-DCs (red dashed lines) based on surface CD32b expression. **(B, C)** Time-course quantification of total live cells and GM-DCs within BM culture. **(C)** CD32b^+^ GM-DC ratio is presented as the proportion of CD32b^+^ GM-DCs within the total GM-DCs. Each dot represents one well of cultured cells. Data were pooled from three independent experiments (n = 12–22 replicates per condition). **(D, E)** Quantification of **(D)** CD32b^-^ and **(E)** CD32b^+^ GM-DCs derived from sorted CD45.2^+^ MDPs or GMPs co-cultured with CD45.1^+^ BM fillers and GM-CSF. Sorted CD45.2^+^ progenitors (5×10² cells) were seeded with CD45.1^+^ BM filler cells (5×10^4^ cells) in 48-well plates. Each dot represents one well. Data were pooled from two independent experiments (n = 5–12 replicates per condition). Error bars indicate median with interquartile range (IQR).

Analysis of the BM cultured with GM-CSF revealed clear kinetics for CD32b expression. Initially, the culture consisted mainly of CD32b^-^ GM-DCs, but the frequency of CD32b^+^ GM-DCs steadily increased over time (**Figures 4A–C**). Thus, the emergence kinetics of the CD32b^-^ GM-DC population paralleled those of MDP GM-DCs, while the kinetics of the CD32b^+^ GM-DC population aligned with those of GMP GM-DCs (**Figures 1C–E**). Indeed, the two progenitor-derived populations showed a clear phenotypic split: MDP GM-DCs were predominantly CD32b^-^, whereas GMP GM-DCs were largely CD32b^+^ (**Figures 4D, E**). Thus, CD32b expression directly reflects the origin of GM-DCs developed in this culture system.

Both MDP- and GMP-derived GM-DCs exhibited the characteristic morphology of DCs, featuring prominent dendrites (**Supplementary Figures 8A, B**). Having confirmed their identity as typical DCs, we then profiled the surface marker expression of CD32b^-^ and CD32b^+^ GM-DCs by flow cytometry to compare their phenotypes. For this analysis, day 7 MDP GM-DCs were used as the representative CD32b^-^ population, while day 21 GMP GM-DCs represented the CD32b^+^ population. The two groups expressed comparable levels of co-stimulatory (CD80, CD86) and maturation (CD83, PD-L2) markers, along with other surface molecules like CD24, CD172a, and CLEC12A (**Supplementary Figures 8C, D**). In contrast, day 21 GMP GM-DCs exhibited significantly higher expression of CD301b. While flow cytometry also detected a slight increase in DEC205 expression on day 21 GMP GM-DCs, this finding was not corroborated by RNA-seq data.

### TNF-α drives the late-stage expansion of GM-DCs in BM culture

We employed Immune Dictionary™ (36), a tool that infers the upstream cytokine landscape from gene expression profiles, to identify the cytokines driving the different developmental kinetics between the two GM-DC populations in BM culture. A comparison of CD32b^+^ (day 21 MDP, day 21 GMP, and day 21 BM) GM-DCs versus CD32b^-^ (day 7 MDP and day 7 BM) GM-DCs revealed 34 DEGs with high expression, which were then used for Immune Dictionary™ calculations (**Supplementary Figure 9A**). The results suggested that IL-21 and TNF-α might have functioned as upstream cytokines specific to the day 21 CD32b^+^ GM-DC populations, but not their day 7 CD32b^-^ counterparts.

To test this, we conducted neutralization assays for IL-21 and TNF-α in BM cultures with GM-CSF. Unlike the anti-IL-21 antibody, which showed only a minor effect, the anti-TNF-α antibody significantly reduced both the number and fraction of GM-DCs in day 21 BM cultures (**Supplementary Figures 9B, C**). Analysis of the BM culture with GM-CSF revealed that the concentration of TNF-α was markedly lower on day 14 relative to days 7 and 21 (**Supplementary Figure 9D**). Despite equivalent levels of TNF-α, genes associated with the “positive regulation of TNF production” were generally more highly expressed in day 21 GM-DCs than in day 7 GM-DCs (**Supplementary Figure 9E**). The addition of TNF-α to BM culture with GM-CSF resulted in a significant, dose-dependent increase in the number and fraction of GM-DCs specifically on day 21, an effect not seen on days 7 and 14 (**Supplementary Figure 9F**). Therefore, it is probable that the influence of TNF-α on the proliferation and/or viability of GM-DCs and their precursors are limited to the later phase of BM culture.

### Pre-GM-DC heterogeneity and TNF-α–driven specification of CD32b^+^ GM-DCs

**Figure 5:**
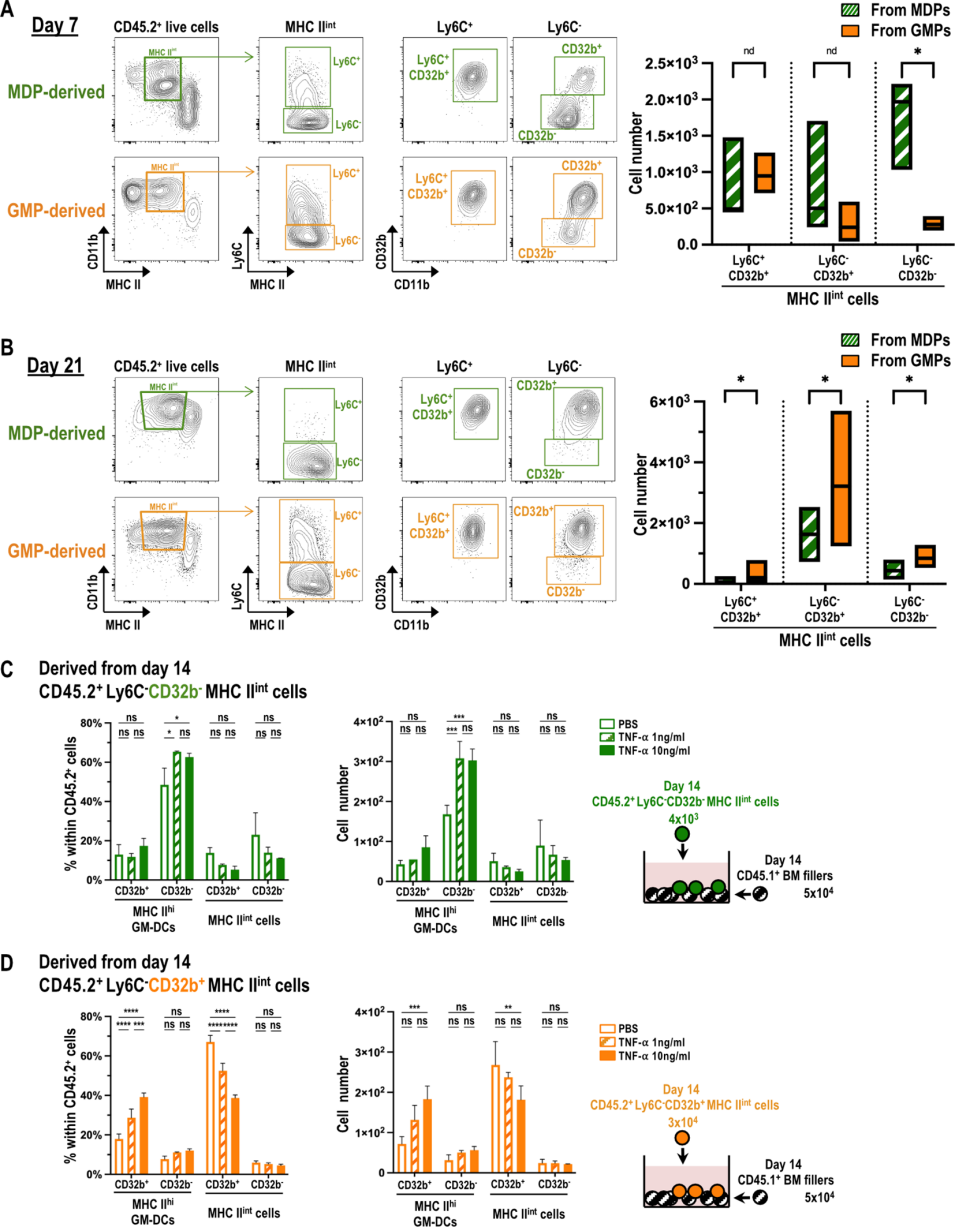
A distinct CD32b^+^ pre-GM-DC subset gives rise to mature CD32b^+^ GM-DCs in a TNF-α-dependent manner. **(A, B)** Identification and quantification of MHC II^int^ populations in BM culture with 3% GM-CSF-conditioned medium. Three subsets were identified within MHC II^int^ cells on **(A)** day 7 MDP- or **(B)** day 21 GMP-derived cultures based on Ly6C and CD32b expression (Ly6C^+^CD32b^+^, Ly6C^-^CD32b^+^, and Ly6C^-^CD32b^-^). Data are representative of three independent experiments, with MDPs performed in triplicate and GMPs performed in sextuplicate. Statistical significance was determined by multiple Mann-Whitney U tests, and p-values were adjusted using the Bonferroni correction for multiple comparisons. Error bars indicate median with interquartile range (IQR). nd, not a discovery; *p ≤ 0.05. **(C, D)** Differentiation of sorted MHC II^int^ subsets. **(C)** Ly6C^–^CD32b^–^ and **(D)** Ly6C^–^CD32b^+^ MHC II^int^ cells were sorted from day 14 BM cultures and co-cultured for 3 additional days with day 14 BM fillers in the presence of the indicated concentrations of TNF-α. Sorted CD45.2^+^ Ly6C^-^CD32b^-^ MHC II^int^ cells were seeded at a density of 4×10^3^ cells and sorted CD45.2^+^ Ly6C^-^CD32b^+^ MHC II^int^ cells were seeded at a density of 3×10^4^ cells. Data are representative of two independent experiments performed in triplicate. Statistical significance was determined using a two-way ANOVA followed by Tukey’s multiple comparison test. Error bars represent mean ± SD. ns, not significant; *p ≤ 0.05; **p ≤ 0.01; ***p ≤ 0.001; ****p ≤ 0.0001.

Sustained generation of MHC II^hi^ GM-DCs for over 21 days in BM culture with GM-CSF indicated a continuous supply of their precursors. This led us to investigate the MHC II^int^ cell population as the likely pre-GM-DCs. We designated this fraction as “pre-GM-DCs” to align with the established DC ontogeny, as the MHC II^int/-^ pool has been shown to contain key intermediates such as the classical “pre-DCs” (37) and the recently identified MHC II^+^ “pre-FL-DCs” (38) Despite being identified as macrophages or “GM-Macs” by Helft and colleagues (24), the MHC II^int^ cells in BM culture with GM-CSF remained poorly characterized. Phenotypic analysis by flow cytometry revealed that Ly6C and CD32b expression levels distinguished MHC II^int^ cells derived from MDPs versus those from GMPs following culture with GM-CSF. On day 7, MDPs primarily differentiated into Ly6C^-^CD32b^-^MHC II^int^ cells, in contrast to GMPs (**Figure 5A**). Conversely, by day 21, GMPs predominantly yielded Ly6C^-^CD32b^+^MHC II^int^ cells (**Figure 5B**). These findings suggested that Ly6C^-^CD32b^-^MHC II^int^ and Ly6C^-^CD32b^+^MHC II^int^ populations may serve as candidate precursors to the CD32b^-^ and CD32b^+^ GM-DC populations, respectively, consistent with their overlapping patterns of emerge (**Figures 4D, E**).

To test this, we isolated Ly6C^−^CD32b^−^ and Ly6C^−^CD32b^+^ subsets from MHC II^int^ cells in day 14 CD45.2^+^ BM cultures and co-cultured them with CD45.1^+^ BM filler cells in the presence or absence of TNF-α (**Supplementary Figure 10A**). While Ly6C^−^CD32b^−^MHC II^int^ cells efficiently produced CD32b^−^ GM-DCs regardless of TNF-α (exhibiting 50~60% differentiation efficiency), Ly6C^−^CD32b^+^MHC II^int^ cells showed a significantly lower spontaneous differentiation baseline (<20%). However, upon TNF-α treatment, this CD32b^+^ subset displayed a more pronounced differentiation response, increasing to approximately 40% (**Figures 5C, D****;** **Supplementary Figures 10B, C**). Although minor cross-contamination may occur due to the inherent limitations of CD32b-based sorting purity, these results indicate that the observed differentiation patterns effectively delineate lineage-specific developmental trajectories.

Meanwhile, in parallel experiments, Ly6C^+^CD32b^+^MHC II^int^ cells failed to differentiate into GM-DCs, irrespective of TNF-α (**Supplementary Figure 10D**). Therefore, these results suggest that Ly6C^-^CD32b^-^MHC II^int^ and Ly6C^-^CD32b^+^MHC II^int^ cells in GM-CSF BM cultures represent the developmental intermediates for CD32b^-^ and CD32b^+^ GM-DCs, respectively, with the latter process potentially modulated by TNF-α. The observed reduction of certain cell populations with increasing concentrations of TNF-α, particularly in the cultures of the Ly6C^−^CD32b^−^ and Ly6C^+^CD32b^+^ subsets, may reflect their significant differentiation into macrophages. Because macrophages adhere strongly to culture plastic, they would naturally be excluded during standard flow cytometric harvesting and analysis (**Figure 5C****;** **Supplementary Figure 10D**).

### Functional specialization of GM-DCs reveals the potent antigen-presenting function of the CD32b^+^ subset

**Figure 6:**
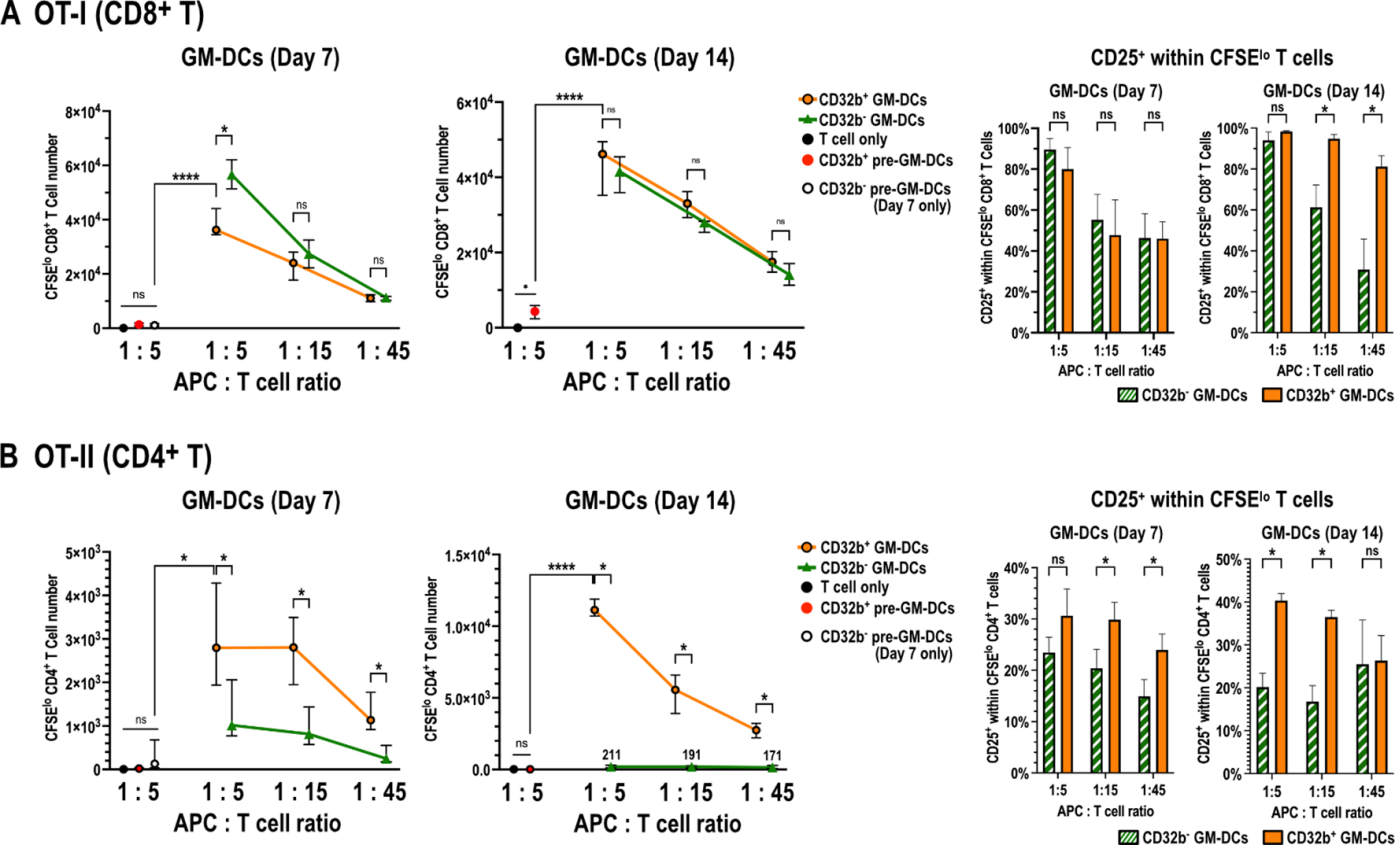
CD32b^+^ GM-DCs exhibit a superior capacity to stimulate CD4^+^ T cells compared to their CD32b^-^ counterparts. **(A)** OT-I (CD8^+^) T cell stimulating capacity of GM-DC subsets sorted on day 7 or day 14. CFSE-labeled OT-I T cells were seeded at 5×10^4^ cells/well and co-cultured for 3 days with sorted CD32b^+^ or CD32b^-^ GM-DCs at the indicated DC-to-T cell ratios. Data are representative of three independent experiments, with CD32b^-^ GM-DCs performed in sextuplicate and CD32b^+^ GM-DCs performed in triplicate. **(B)** OT-II (CD4^+^) T cell stimulating capacity of GM-DC subsets sorted on day 7 or day 14. CFSE-labeled OT-II T cells were seeded at 5×10^4^ cells/well and co-cultured for 4 days with sorted CD32b^+^ or CD32b^-^ GM-DCs at the indicated DC-to-T cell ratios. Line graphs display the total number of proliferated (CFSE^lo^) T cells, and bar graphs show the frequency of activated (CD25^+^) T cells within this population. Data are representative of two independent experiments performed in triplicate. Statistical significance was determined by multiple Mann-Whitney U tests, and p-values were adjusted using the Bonferroni correction for multiple comparisons. Error bars indicate median with interquartile range (IQR). ns, not significant; *p ≤ 0.05; **p ≤ 0.01; ***p ≤ 0.001; ****p ≤ 0.0001.

To assess antigen presentation at different developmental stages, we isolated ovalbumin (OVA)-pulsed CD32b^-^ and CD32b^+^ GM-DCs from BM cultures on days 7 and 14. These isolated cells were then co-cultured with OVA-specific transgenic T cells from either OT-I (CD8^+^) or OT-II (CD4^+^) mice. While day 7 CD32b^-^ GM-DCs were more potent stimulators of CD8^+^ OT-I T cells than their CD32b^+^ counterparts, this dynamic changed by day 14, when CD32b^+^ GM-DCs demonstrated similar capacity to induce their proliferation and slightly better CD25 expression than CD32b^-^ GM-DCs (**Figure 6A**; **Supplementary Figures 11A, C**). In contrast, the CD32b^+^ GM-DCs were consistently superior in activating CD4^+^ OT-II T cells, driving robust proliferation and CD25 expression at both time points. The capacity of the CD32b^-^ GM-DCs to stimulate CD4^+^ OT-II T cells, however, was weak on day 7 and virtually absent on day 14 (**Figure 6B**; **Supplementary Figures 11B, C**).

To investigate the molecular mechanisms underlying the different antigen presentation capacities of CD32b^-^ and CD32b^+^ GM-DCs, we performed a Gene Ontology Biological Process (GO: BP) enrichment analysis on the DEGs between day 7 MDP-derived and day 21 GMP-derived GM-DCs. The top 20 most enriched GO terms included numerous pathways related to lymphocyte and leukocyte activation and proliferation (**Supplementary Figure 12A**). A subsequent heatmap analysis revealed a distinction between the subsets: GMP-derived GM-DCs expressed higher levels of genes such as *CD28*, *CD6*, *CD24A*, *CD209A*, and *TNFSF13*, while MDP-derived GM-DCs showed elevated expression of genes such as *IDO1*, *IRF1*, *NRARP*, *IL2RA*, and *CD40* (**Supplementary Figure 12B**).

### Divergent differentiation of GMPs and MDPs into distinct DC subsets driven by GM-CSF *in vivo*

In our analysis, we followed the conventional classification of tissue DCs, where MHC II^+^CD11c^+^ cells are divided into CD11b^−^ DC1 and CD11b^+^ DC2 subsets (39). We then further subdivided the DC2 compartment into CD32b^+^ and CD32b^−^ populations to investigate their respective heterogeneity. We investigated the *in vivo* impact of systemic GM-CSF treatment on DC populations across various murine tissues (**Supplementary Figure 13A**). Although this treatment did not significantly alter total CD45^+^ cell counts, it increased the overall DC compartment (**Supplementary Figures 13B, C**). Notably, it drove a profound, tissue-dependent expansion of the CD32b^+^ GM-DC2 subset with distinct kinetics (**Supplementary Figures 13D, E**). In the BM, the increase was transient, peaking at day 4. In contrast, the expansion was sustained and progressive in peripheral organs like the liver, lung, and spleen, where CD32b^+^ GM-DC2s were virtually absent in control mice. By day 7, the effect was most pronounced in the liver and lung, where newly emerged CD32b^+^ GM-DC2s became the dominant DC subset and comprised over 20% of all CD45^+^ cells. The spleen, however, showed a unique response by expanding both CD32b^+^ and CD32b^-^ GM-DC2 subsets (**Supplementary Figure 13E**).

**Figure 7:**
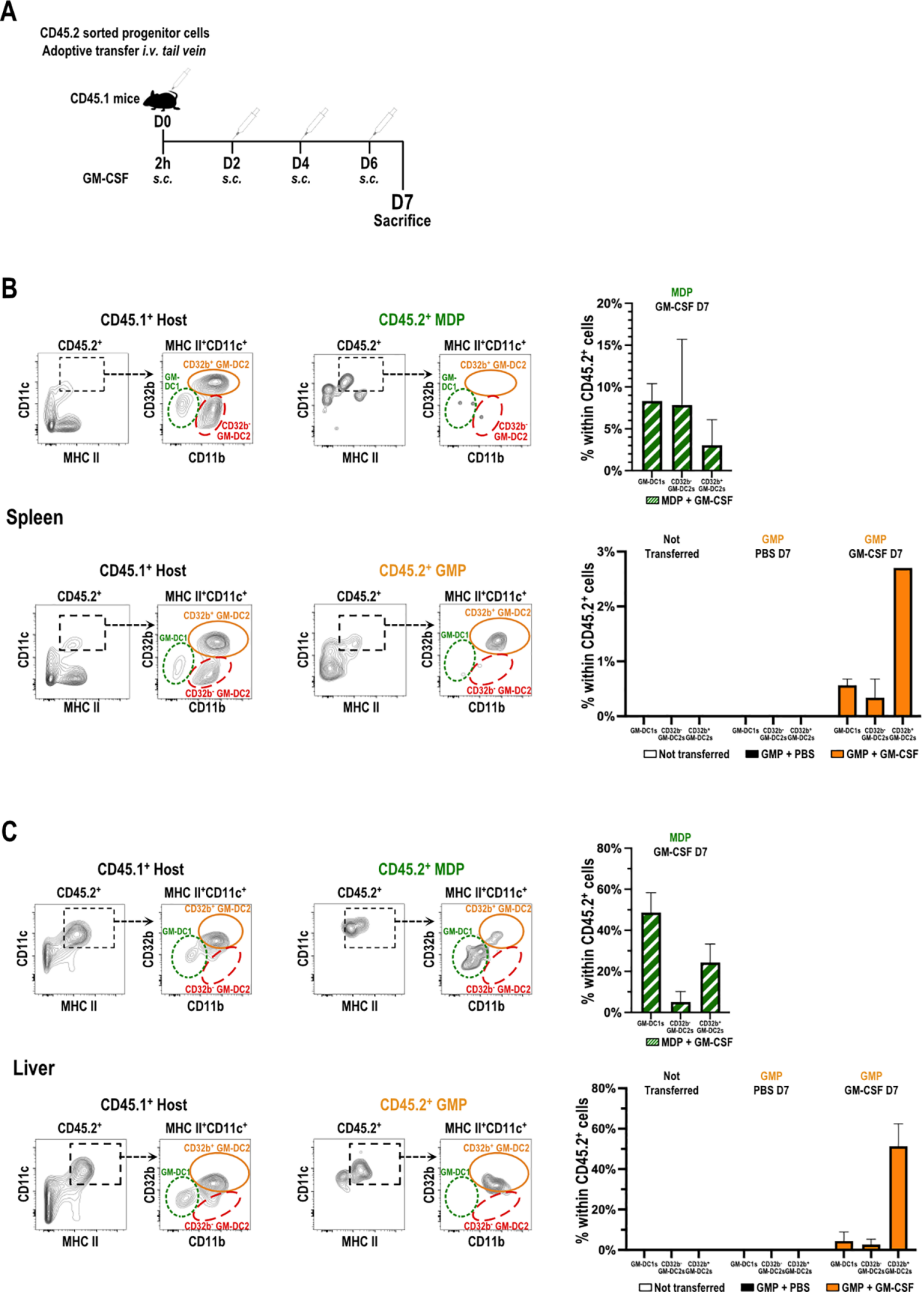
GMPs generate CD32b^+^ GM-DCs with slower kinetics than MDPs *in vivo*. **(A)** Scheme of the adoptive transfer experiment. A total of 1×10^4^ to 4×10^4^ sorted CD45.2^+^ GMPs or MDPs were transferred into CD45.1^+^ recipient mice, which were then subcutaneously injected with 10 µg of recombinant mouse GM-CSF every other day. Organs were harvested on day 7 for analysis. **(B, C)** Representative flow cytometry plots and quantification of donor-derived GM-DC subsets in the **(B)** spleen and **(C)** liver on day 7. Donor cells were identified by gating on CD45.2^+^ cells. Within the MHC II^+^CD11c^+^ GM-DC population, GM-DC1s were defined as CD11b^-^, while CD11b^+^ cells were further subdivided into CD32b^-^ and CD32b^+^ GM-DC2 subsets. Data are representative of two independent experiments with two biological replicates per group. Error bars represent mean ± SD.

In parallel experiments tracking adoptively transferred GMPs in GM-CSF-treated mice, the number of GMP-derived cells and DCs peaked in the BM and spleen at day 7 post-transfer (**Supplementary Figure 14**). Further experiments in GM-CSF-treated mice demonstrated that MDPs and GMPs displayed divergent differentiation patterns at day 7 post-adoptive transfer (**Figure 7A**). MDPs produced a broader range of GM-DC subsets, including CD11b^-^ GM-DC1s and CD32b^-^ GM-DC2s, while GMPs predominantly gave rise to CD32b^+^ GM-DC2s across the tissues examined (**Figures 7B, C**; **Supplementary Figure 15**). These results suggest GMPs as the major source of the expanding CD32b^+^ GM-DC2 pool that occurs *in vivo* during conditions of elevated GM-CSF, such as infection and inflammation (13, 40, 41). Analysis of GM-DC subsets in the spleen and liver revealed that MDP-derived cells differentiated more rapidly and were thus more abundant at day 4 post-transfer, whereas GMP-derived GM-DCs made a more significant contribution at day 7 post-transfer (**Figures 7B, C**; **Supplementary Figures 16A-C**).

In alignment with observations from *in vitro* BM cultures, these results show that *in vivo*, high GM-CSF levels drive GMPs to slowly generate CD32b^+^ GM-DC2s, while MDPs quickly produce a diverse array of DC subtypes.

## Discussion

The cellular origin of GM-DCs has been a subject of debate. Yanez and colleagues proposed that MDPs, rather than GMPs, were the primary source of DCs during inflammation (15). This model was supported by fate-mapping studies using an *MS4A3*-based system, which appeared to exclude GMP-derived GM-DCs (16). This reinforced the classical view that GM-DCs originate from monocytes descending from MDPs and common monocyte progenitors (cMoPs), a concept consistent with early *in vitro* methods for generating human MoDCs (9, 24). However, more recent evidence has challenged this view. Lutz and colleagues showed that GMPs are the primary drivers of myelopoiesis in BM cultures with GM-CSF (14). Furthermore, Rodrigues and colleagues reported that the GMP-specific marker *MS4A3* is also present in Ly6C^+^ MDPs (42), which highlights the difficulty of using certain markers to definitively distinguish between myeloid progenitors. Ultimately, the current body of research suggests that both MDPs and GMPs likely contribute to GM-DC development in a context-dependent manner.

Our study has clarified the ambiguity surrounding GM-DCs generated in BM cultures. We demonstrate that this common *in vitro* system yields not a single, homogenous population but a heterogeneous mixture of two distinct GM-DC subsets originating from separate myeloid progenitors, i.e., MDPs and GMPs, and are distinguished by their expression of CD32b. The CD32b^-^ GM-DCs develop rapidly from MDPs and their derivatives (e.g., CDPs), while the CD32b^+^ GM-DCs differentiate more slowly from GMPs and their derivatives (e.g., MoPs). This temporal difference in development correlates with their T cell-stimulating capacity; while both subsets activate CD8^+^ T cells with similar efficiency, CD32b^+^ GM-DCs are superior in stimulating CD4^+^ T cells. Although our DEG analysis identified distinct transcriptomic heterogeneity between the two subsets, we also observed the presence of lineage-atypical transcripts. Consequently, these DEG findings represent an initial exploratory analysis; further validation, including their protein expression levels or specific functional assays, is required in future studies to clarify the biological significance of these GM-DC subset-specific gene signatures. Therefore, these results serve as a foundational profile that warrants more rigorous functional validation to determine their precise biological impact.

Furthermore, the immediate precursors for the CD32b^-^ and CD32b^+^ GM-DCs are identified as Ly6C^-^CD32b^-^ and Ly6C^-^CD32b^+^ MHC II^int^ cells, respectively. It is likely that the CD11c^+^MHC II^int^ fraction represents a heterogeneous state where pre-GM-DCs coexist with precursors of other lineages, such as M1-like macrophages (43, 44). By employing a selective harvesting procedure to exclude adherent macrophages, we confirmed that this population possesses a robust potential to differentiate into DCs expressing specific maturation markers, such as CD83 and CD86. Therefore, while current markers have technical limitations in achieving absolute isolation, this mixed population undeniably harbors progenitors with robust DC differentiation potential. Accordingly, the term “pre-GM-DC” is used here not to claim a strictly homogeneous population, but to emphasize the developmental commitment toward the DC lineage within this MHC II^int^ fraction. In this light, our findings clarify that the heterogeneous MHC II^int^ population, previously termed “GM-Macs” (24), is composed of pre-GM-DCs or immature DCs, as proposed by Lutz and colleagues (28).

In addition, we discovered that the delayed emergence of CD32b^+^ GM-DCs is driven by two linked factors. First, TNF-α is a pleiotropic cytokine that drives both the early differentiation of DCs (by synergizing with GM-CSF) and the terminal activation and maturation of immature DCs; moreover, recent studies demonstrate that TNF-α acts as a critical factor in determining differentiation into specific DC subsets (45). In this context, unlike their CD32b^-^ counterparts, CD32b^+^ pre-GM-DCs appear to benefit significantly from TNF-α stimulation to differentiate more efficiently into CD32b^+^ GM-DCs. Second, TNF-α levels in the BM cultures with GM-CSF follow a distinct temporal pattern: they are initially high at day 7, become nearly undetectable by day 14, and then surge to their highest levels by day 21. This late surge indicates that it may provide a favorable environment or a supportive signal for the delayed emergence of CD32b^+^ GM-DCs.

Our prior work demonstrated the slow generation of DCs from splenocytes cultured with GM-CSF (33). This delayed kinetic resembles the emergence of CD32b^+^ GM-DCs observed in BM cultures with GM-CSF. Consistent with the spleen’s notable lack of MDPs, we now identified GMPs as the major source of GM-DCs in splenocyte cultures. Bulk RNA-seq analysis further supported this finding, showing that splenic GM-DCs are CD32b^+^ and cluster more closely with CD32b^+^ GM-DCs from BM GMPs than with their CD32b^-^ MDP-derived counterparts. These findings suggest that GMPs from the spleen and the BM may be largely indistinguishable.

Meanwhile, adoptive transfer of myeloid progenitors into GM-CSF-treated mice demonstrated that MDPs and GMPs contribute distinctly and in a time-dependent manner to *in vivo* GM-DC development, mirroring the patterns observed *in vitro*. While adoptively transferred MDPs rapidly generate both GM-DC1 and GM-DC2 subsets, GMPs differentiate more slowly and selectively, primarily yielding the CD32b^+^ GM-DC2 population, the most abundant DC subset in the spleen, liver, and lung of GM-CSF-treated mice. This finding aligns with our previous study, in which we identified that daily GM-CSF injections generate a novel DC subset, GM-CSF-induced DCs (GMiDCs), that becomes the dominant DC population in the mouse spleen after 3 days (13). Therefore, the present study refers to GMiDCs as CD32b^+^ GM-DC2s. All in all, CD32b^+^ GM-DCs from *in vitro* cultures are phenotypically and functionally similar to splenic GMiDCs from GM-CSF-treated mice, as both cell types exhibit comparable surface marker profiles, including CD32b, and are more potent stimulators of CD4^+^ T cells than other DC subsets (13).

Previous work demonstrated that GMiDCs differentiate from monocytes *in vivo* (13). In our *in vitro* BM cultures, we identified a population of pre-GM-DCs that share phenotypic markers with classical monocytes such as CD11b^+^ and CD115^+^. However, we deliberately avoided the extensive use of the term “monocytes” for this population, opting instead for a marker-based description to maintain taxonomic precision. This approach is informed by recent advances in DC ontogeny, particularly regarding DC3s, which have identified specific progenitor populations that share nearly identical markers with monocytes (e.g., CD11b^+^, CD115^+^, and CD14^+^) yet represent a distinct lineage separate from classical monocytes (17). By adhering to this stringent classification, we aim to avoid oversimplifying the identity of these precursors within the GM-CSF BM culture system. While conventional mouse models have long emphasized the MDP-derived pathway as the primary source of MoDCs (15), our findings reflect a specific developmental trajectory toward the DC lineage that is distinct from the classical MDP-derived monocyte pathway. This suggests the possibility that the GMP lineage serves as a parallel source of a specialized, monocyte-like pool required for the generation of GMiDCs *in vivo*.

Furthermore, our splenocyte cultures with GM-CSF showed a slow generation of GM-DCs from GMPs, rather than an early generation from the abundant splenic monocytes (46). This observation is consistent with previous findings that GM-DC expansion in BM cultures is driven by MoPs and higher progenitors, not by their immediate monocyte precursors (24). The selective presence of GMPs, but not MDPs, in the spleen also suggests GMPs, but not MDPs, can circulate from the BM and differentiate in peripheral tissues.

In addition to resembling GMiDCs, the CD32b^+^ GM-DCs share key characteristics with the recently identified DC3 subset, including shared surface markers and responsiveness to GM-CSF (4). While current models trace DC3 development primarily through the MDP to Ly6C^+^ MDP pathway (17), our findings reveal a parallel route involving MoPs derived from GMPs. The close phenotypic similarity between intermediate Ly6C^+^ GMPs and Ly6C^+^ MDPs may have led to these populations being conflated in previous analyses (42).

Therefore, CD32b^+^ GM-DCs may represent a converging or functionally related subset within the broader DC3 spectrum. In this context, while recent studies have identified markers such as *Ms4a3, Cd177*, and *Slamf7* as instrumental for tracking GMP- or MDP-derived monocyte lineages (16), our data suggest these markers may not remain reliable indicators for terminal GM-DC populations (data not shown). Specifically, the low or non-differential expression of these genes in our system indicates that their signature may diminish or equalize upon terminal differentiation. Consequently, the robust and consistent expression of CD32b observed in our study highlights its potential as a more definitive and stable marker for distinguishing these mature subsets compared to previously established intermediate markers.

In support of this model, a recent study by Amon et al. identified a clear developmental bifurcation of *in vivo* BM GM-DCs into CD103^+^ cDC1 and CD301b^+^ cDC2 lineages through single-cell RNA sequencing (47). These *in vivo* findings closely parallel the lineages identified in our *in vitro* culture system; specifically, our CD32b^-^ GM-DCs express CD103^+^, while the CD32b^+^ GM-DCs are characterized by CD301b^+^. Crucially, CD32b^+^ GM-DCs exhibit a superior capacity to stimulate OT-II T cells compared to the CD32b^-^ GM-DCs, further aligning them with the cDC2 phenotype. These parallels are highly complementary to our work, providing critical *in vivo* evidence that validates the physiological relevance of our *in vitro* developmental model.

In conclusion, this study clarifies the developmental origins of GM-DCs and establishes CD32b as a discriminating marker of their heterogeneity. We demonstrate that GM-DCs arise from two distinct progenitor pathways, MDP-CDP and GMP(MDP)-MoP, resulting in subsets with differing differentiation kinetics and T cell-stimulating capacities (**Supplementary Figure 18**). The contribution of GMPs to the development of CD32b^+^ GM-DCs has been largely overlooked, and our findings provide new insight into the ontogeny of emerging subsets like GMiDCs and DC3s. Notably, our study addresses the translational significance of these findings by identifying a functional GMP-derived DC pathway in mice, thereby bridging a persistent gap with human myeloid DC ontogeny. While it is well-established that human DCs can differentiate from GMP-derived monocytes (48), our work reveals a significant parallel by demonstrating the existence and characteristics of this lineage in mice. We believe that this more comprehensive model better aligns mouse data with human biology, improving the clarity and directness with which mouse findings can be applied to human immunology. This offers a robust and translatable framework for future research on the functional and therapeutic applications of human MoDCs. Altogether, this work refines the current model of DC lineage diversification under GM-CSF and provides a framework for investigating inflammation-associated DC subsets in both experimental and physiological contexts. Future *in vivo* studies are required to fully delineate these lineage relationships.

## Data Availability

The datasets presented in this study can be found in online repositories. The names of the repository/repositories and accession number(s) can be found in the Materials and Methods section and/**Supplementary Material**.

## Supplementary material

Supplementary Figure 1 Time-course analysis of splenocyte cultures with GM-CSF and *in vivo* quantification of myeloid progenitor cell populations. **(A)** Representative flow cytogram and cell number of time-course culture of splenocytes with 1% GM-CSF-conditioned medium. Splenocytes were seeded at a density of 1×10^6^ cells in 24-well plate. Representative data are shown from three independent experiments in quadruplicate. **(B)** Number of total lives and myeloid progenitors (defined as Lineage(Lin)^-^CD117^+^) in BM and spleen. Lin includes Sca-1 and CD127 for excluding non-myeloid progenitors, i.e. common lymphoid progenitors (CLPs), multipotent progenitors (MPPs) and hematopoietic stem cells (HSCs). Data are shown from three independent experiments with biological triplicates using three individual mice. Error bars indicate mean ± SD. ns, not significant; *, p ≤ 0.05; **, p ≤ 0.01; ***, p ≤ 0.001; ****, p ≤ 0.0001.

Supplementary Figure 2 Marker-based flow cytometric identification of myeloid progenitors in BM and spleen. (A-B) Gating strategy and quantitative distribution of myeloid progenitors from **(A)** BM and **(B)** spleen by flow cytometry. Lin of myeloid progenitors includes Sca-1 and CD127. LSK (defined as Lin^-^Sca-1^+^c-Kit^+^) includes HSC and MPP; FLT3^+^ CMP; FLT3^+^ Common Myeloid Progenitor defined as Lin^-^c-Kit^+^CSF-1R^-^CD16/32^-^CD34^+^Ly6C^-^FLT3^+^, FLT3^-^ CMP; FLT3^-^ Common Myeloid Progenitor defined as Lin^-^c-Kit^+^CSF-1R^-^CD16/32^-^CD34^+^Ly6C^-^FLT3^-^, GMP; Granulocyte Monocyte Progenitor defined as Lin^-^c-Kit^+^CSF-1R^-^CD16/32^+^CD34^+^Ly6C^-^FLT3^-^, Ly6C^+^ GMP; Ly6C^+^ GMP defined as Lin^-^c-Kit^+^CSF-1R^-^CD16/32^+^CD34^+^Ly6C^+^FLT3^-^, MEP; Megakary-ocyte Erythrocyte Progenitor defined as Lin^-^c-Kit^+^ CSF-1R^-^CD16/32^-^CD34^-^, MDP; Macrophage Dendritic cell Progenitor defined as Lin^-^c-Kit^+^CSF-1R^+^CD16/32^-^CD34^+^Ly6C^-^FLT3^+^, MoP; Monocyte Progenitor defined as Lin^-^c-Kit^+^CSF-1R^+^CD16/32^+^CD34^+^Ly6C^+^FLT3^-^, CDP; Common Dendritic cell Progenitor defined as Lin^-^c-Kit^int^CSF-1R^+^FLT3^+^. Representative data are shown from five biologically independent samples (n=5).

Supplementary Figure 3 Gating strategy of splenic myeloid progenitors and representative flow cytograms of CD45.1^+^ splenocyte fillers. **(A)** Gating strategy and cell purity of sorted splenic GMPs and FLT3^-^ CMPs. **(B)** Representative flow cytograms of splenocyte fillers in **Figure 1**, **(A, B)**. CD45.1^+^ splenocyte fillers were seeded at a density of 1×10^6^ cells in 24-well plate.

Supplementary Figure 4 Co-culture conditions of CD45.1^+^ BM fillers and gating strategy for BM GMPs and MDPs. **(A)** Gating strategy and cell purity of sorted BM GMPs and MDPs. **(B)** Representative flow cytograms of BM fillers in **Figure 2A**. CD45.1^+^ BM fillers were seeded at a density of 5×10^4^ cells and CD45.2^+^ sorted BM progenitor cells were seeded at a density of 5×10^2^ cells in 48-well plate. **(C)** Graphical scheme and representative flow cytograms of simultaneous co-culture experiment of BM fillers, BM MDPs and GMPs. CD45.2^+^ and CD45.1^+^ sorted progenitors were co-seeded at a density of 5×10^2^ cells, together with 5×10^4^ CD45.1^+^ BM filler cells, in 48-well plates.

Supplementary Figure 5 Distinct progenitor subsets follow different GM-DC differentiation kinetics from culture without BM fillers. **(A)** Representative flow cytogram and cell number of time-course culture of BM GMPs and BM MDPs without BM fillers under 5% GM-CSF-conditioned medium. Sorted cells were seeded at a density of 1×10^3^ cells in 24-well plate. Representative data are shown from 3 independent experiments in triplicate. **(B)** Cell number and proportion of time-course culture of BM GMPs and Ly6C^+^ GMPs without BM fillers under 5% GM-CSF-conditioned medium. Sorted cells were seeded at a density of 5×10^2^ cells in 48-well plate. Representative data are shown from 3 independent experiments in quadraplicate. **(C)** Representative flow cytogram and cell number of time-course culture of BM GMPs and BM MDPs with twofold higher cell numbers. Sorted CD45.2^+^ progenitor cells were seeded at a density of 1×10^3^ cells and BM filler cells were seeded at a density of 1x10^5^ cells in 24-well plate. Representative data are shown from 3 independent experiments in triplicate. Error bars indicate mean ± SD across multiplicate samples.

Supplementary Figure 6 Flow cytometric analysis of BM myeloid progenitors under short-term GM-CSF culture conditions. **(A)** Representative flow cytograms and quantification of myeloid progenitors from Lin^-^Sca-1^-^CD127^-^CD117^+^ enriched BM cells cultured with 3% GM-CSF-conditioned medium for 0, 1 or 3 days. Enriched cells were seeded at a density of 2×10^4^ cells and cultured for 1 or 3 days in 24-well plates. **(B–E)** Representative flow cytograms and bar graphs showing the differentiation outcomes of sorted **(B)** FLT3^+^ CMPs, **(C)** FLT3^-^ CMPs, **(D)** MDPs, and **(E)** GMPs after 1 or 3 days of culture with GM-CSF. Each progenitor population was seeded at a density of 3×10^3^-5×10^3^ cells and cultured for 1 or 3 days in 48-well plates. Data shown from three independent experiments performed in duplicate. Error bars indicate mean ± SD.

Supplementary Figure 7 Transcriptomic and phenotypic characterization of developmentally distinct MDP- and GMP-derived GM-DC subsets. **(A)** Flow cytometric validation of selected surface markers identified in DEG analysis. Normalized Mean fluorescence index (MFI) values of four representative markers from each subset are shown. Normalized MFI was calculated by subtracting the isotype control mean fluorescence from the sample mean fluorescence, and then dividing the result by the isotype control mean. Representative flow cytometric histograms are shown with isotype controls overlaid in gray. Representative data are shown from two independent experiments. **(B)** Principal component analysis (PCA) of bulk RNA-seq data from GM-DCs sorted at day 7 and day 21. DCs were collected from splenocyte cultures (day 21), BM cultures (day 7 and 21) and from cultures of sorted MDPs (day 7 and day 21) or GMPs (day 21) under GM-CSF conditions. **(C)** Volcano plot of bulk RNA-seq data comparing MDP-derived GM-DCs (day 7) and GMP-derived GM-DCs (day 21). Selected genes (*FCGR2B*, *FCGR3*) are highlighted with blue dot. **(D)** Representative Flow cytograms of GMP-derived cells cultured with GM-CSF. Overlaid black histograms represent the fluorescence-minus-one (FMO) controls.

Supplementary Figure 8 Morphology and surface marker expression of MDP GM-DCs (day 7) and GMP GM-DCs (day 21). **(A, B)** Representative phase-contrast micrographs showing the morphology of **(A)** MDP GM-DCs at day 7 and **(B)** GMP GM-DCs at day 21 of culture. Images were captured at 200x magnification. Scale bars: 50 μm. **(C, D)** Flow cytometric histograms of various myeloid markers. Representative flow cytometric histograms are shown with isotype controls overlaid in gray. Representative data are shown from two independent experiments.

Supplementary Figure 9 TNF-α promotes the differentiation of CD32b^+^ GM-DCs. **(A)** Bioinformatic prediction of key cytokines involved in GM-DC differentiation. Genes significantly upregulated in GM-DCs on day 7 versus day 21 (fold change > 4, p < 0.01) were used as input for a cytokine prediction analysis (Immune Dictionary™). Cytokines were ranked based on a normalized score. Normalized score derived from categorical binning of gene-level expression (0 = low, 1 = intermediate, 2 = high). **(B)** Effect of IL-21 neutralization on GM-DC generation. BM cells (1×10^5^) were cultured with 3% GM-CSF-conditioned medium and treated with either PBS or an anti-mouse IL-21 antibody (10 µg/mL). The frequency and number of DCs were assessed on day 21. **(C)** Effect of TNF-α neutralization on GM-DC generation. BM cells (5×10^4^ cells/well) were cultured with 3% GM-CSF-conditioned medium and treated with either PBS or an anti-mouse TNF-α antibody (10 µg/mL). The frequency and number of DCs were analyzed on day 21. **(D)** Quantification of TNF-α in culture supernatants. Supernatants from BM cultures with 3% GM-CSF-conditioned medium (1×10^5^ cells/well) were harvested on days 7, 14, and 21 and analyzed by ELISA. Medium containing only GM-CSF served as the control. Data are from two independent experiments. **(E)** Heatmap of genes associated with the Gene Ontology (GO) term “Positive regulation of TNF production” (GO:0032760), comparing day 7 MDP-derived GM-DCs with day 21 GMP-derived GM-DCs. **(F)** Effect of exogenous TNF-α on BM cultures. BM cells (5×10^4^ cells/well) were cultured with 3% GM-CSF-conditioned medium and treated with the indicated concentrations of recombinant mouse TNF-α. Total cell number, DC frequency, and absolute DC number were quantified on days 7, 14, and 21. Data are representative of two independent experiments performed in triplicate. Statistical significance was determined using a two-way ANOVA followed by Tukey’s multiple comparison test. Error bars represent mean ± SD. ns, not significant; *, p ≤ 0.05; **, p ≤ 0.01; ***, p ≤ 0.001; ****, p ≤ 0.0001.

Supplementary Figure 10 Sorting purity and co-culture of CD32b^+^ and CD32b^-^ MHC II^int^ subsets from BM culture with GM-CSF. **(A)** Sorting purity of pre-DC candidates. CD32b^+^ pre-GM-DC (MHC II^int^Ly6C^-^CD32b^+^) and CD32b^-^ pre-GM-DC (MHC II^int^Ly6C^-^CD32b^-^) populations are sorted from 14-day cultured BM with 3% GM-CSF-conditioned medium. **(B, C)** Representative flow cytograms of co-cultured **(B)** CD32b^+^ and **(C)** CD32b^-^ MHC II^int^ populations with 14-day cultured CD45.1^+^ BM fillers and GM-CSF. **(D)** Representative flow cytograms and quantification of co-cultured Ly6C^+^CD32b^+^ populations with 14-day cultured CD45.1^+^ BM fillers and GM-CSF. Representative flow cytograms are shown. Data are shown from two independent experiments in triplicate. Statistical significance was determined using a two-way ANOVA followed by Tukey’s multiple comparison test. Error bars indicate mean ± SD across triplicate samples. *, p ≤ 0.05; **, p ≤ 0.01; ***, p ≤ 0.001; ****, p ≤ 0.0001.

Supplementary Figure 11 Representative flow cytograms of antigen presentation by GM-DCs in **Figure 6**. **(A, B)** Representative flow cytograms of **(A)** OT-I (CD8^+^) and **(B)** OT-II (CD4^+^) T cell proliferation in response to CD32b^+^ or CD32b^-^ GM-DCs sorted at day 7 or 14. **(C)** Representative flow cytograms of T cell priming assay using pre-GM-DC populations and T cells only. Ly6C^-^CD32b^-^ and Ly6C^-^CD32b^+^ pre-GM-DCs were co-cultured with OT-I (CD8^+^) and OT-II (CD4^+^) T cells at a 1:5 ratio.

Supplementary Figure 12 Transcriptional and functional differences between GM-DCs on day 7 or day 21 revealed by GO analysis. **(A)** Gene ontology: Biological process enrichment analysis (GO: BP) from differentially expressed genes between MDP GM-DCs (day 7) and GMP GM-DCs (day 21). Top 20 enriched terms are shown after size-based filtering. **(B)** Heatmap of genes associated with the activation and proliferation-associated GO terms from **(A)**.

Supplementary Figure 13 Organ-specific kinetics of DC subset generation following *in vivo* GM-CSF administration. **(A)** Scheme of the experimental design. Mice were subcutaneously injected with recombinant mouse GM-CSF, and organs were harvested on day 4 or day 7 for flow cytometric analysis. **(B)** Total number of CD45^+^ cells in the BM, spleen, lung, liver, and skin-draining lymph nodes (sdLNs) on day 4 and day 7 post-injection. **(C)** Percentage of MHC II^+^CD11c^+^ DC populations within CD45^+^ cells in the BM, spleen, lung, liver, and sdLNs on day 4 and day 7 post-injection. **(D)** Representative flow cytogram of MHC II^+^CD11c^+^ DC subsets in various organs on day 7. MHC II^+^CD11c^+^ DC subsets were classified as CD11b^-^ GM-DC1s, CD11b^+^CD32b^-^ GM-DC2s, and CD11b^+^CD32b^+^ GM-DC2s. **(E)** DC subsets within the indicated organs on day 4 and day 7. Data are representative of two independent experiments with two biological replicates per group. Statistical significance was determined using a two-way ANOVA followed by Tukey’s multiple comparison test. Error bars represent mean ± SD. ns, not significant; *, p ≤ 0.05; **, p ≤ 0.01; ***, p ≤ 0.001; ****, p ≤ 0.0001.

Supplementary Figure 14 Kinetics of adoptively transferred GMP-derived DC generation in BM and spleen following GM-CSF treatment. Cell number of CD45.2^+^ GMP-derived cell and MHC II^+^CD11c^+^ DC counts in the BM and spleen following GM-CSF administration at day 4, 7, or 14.

Supplementary Figure 15 Adoptively transferred GMP and MDP in lung at day 7 following GM-CSF treatment. Representative flow cytograms and quantification of donor-derived lung DC subsets at day 7 following PBS or GM-CSF treatment. DCs were defined as MHC II^+^CD11c^+^ cells; CD11b^-^ cells as GM-DC1s, and CD11b^+^ GM-DC2s were further subdivided into CD32b^-^ and CD32b^+^ GM-DC2s. Donor cells were identified by CD45.2^+^ gating. Representative flow cytograms are shown. Error bars indicate mean ± SD across duplicate biological replicates. Data are representative of two independent experiments.

Supplementary Figure 16 Adoptively transferred GMP and MDP in spleen and liver at day 4 following GM-CSF treatment. **(A)** Scheme of the *in vivo* adoptive transfer experiment. Sorted CD45.2^+^ GMPs or MDPs were transferred into CD45.1^+^ recipient mice, which were then treated with GM-CSF. Organs were harvested at day 4 for flow cytometric analysis. **(B)** Representative flow cytograms of splenic and hepatic MHC II^+^CD11c^+^ DC subsets at day 4 following GM-CSF administration. Representative flow cytograms are shown. Data are representative of two independent experiments with two biological replicates per group. **(C)** Representative flow cytograms of donor-derived splenic and hepatic GM-DC subsets on day 4. Donor cells were identified by CD45.2^+^ gating. Representative flow cytograms are shown. Data are representative of two independent experiments with two biological replicates per group. Error bars represent mean ± SD.

Supplementary Figure 17 Validation of GM-DC identification using MHC II and CD11c markers. **(A)** Corresponding flow cytograms to **Supplementary Figure 1A**, showing MHC II versus CD11c expression. **(B)** Corresponding flow cytograms of **Figure 1A**, showing MHC II versus CD11c expression. **(C)** Corresponding flow cytograms of **Supplementary Figure 3B**, showing MHC II versus CD11c expression. **(D)** Corresponding flow cytograms of **Supplementary Figure 4B**, showing MHC II versus CD11c expression. **(E)** Corresponding flow cytograms of **Supplementary Figure 4C**, showing MHC II versus CD11c expression. **(F)** Corresponding flow cytograms of **Supplementary Figure 5A**, showing MHC II versus CD11c expression.

Supplementary Figure 18 Two-track model of GM-DC differentiation trajectories *in vitro*. Schematic summary of the dual developmental pathways leading to GM-DC heterogeneity. The early differentiation track initiates from FLT3-expressing progenitors, including FLT3^+^ CMPs, MDPs, and CDPs, which rapidly give rise to CD32b^−^ pre-GM-DCs and CD32b^−^ GM-DCs within the first week of culture. The late differentiation track arises from FLT3^−^ CMPs, which serve as an intermediate population between FLT3^+^ CMPs and GMPs. This track progresses through CD32b-sharing progenitors such as GMPs, Ly6C^+^ GMPs, and MoPs. The pathway transitions through a CD32b^+^ Ly6C^+^ CD115^+^ CD11b^+^ intermediate state to form CD32b^+^ pre-GM-DCs, resulting in the delayed emergence of CD32b^+^ GM-DCs by Week 3.
