# Correction: CD32b defines distinct dendritic cell lineages generated from the culture of bone marrow with GM-CSF

**DOI:** 10.3389/fimmu.2026.1905985

**Published:** 2026-06-29

**Authors:** Wanho Choi, Seul Hye Ryu, Ji Soo Park, Da Eun Park, Min Kyung Chu, Hye Young Na, Chae Gyu Park

**Affiliations:** 1 Laboratory of Immunology, Severance Biomedical Science Institute, Yonsei University College of Medicine, Seoul, Republic of Korea; 2 Brain Korea 21 FOUR Project for Medical Science, Yonsei University College of Medicine, Seoul, Republic of Korea; 3 Department of Neurology, Severance Hospital, Yonsei University College of Medicine, Seoul, Republic of Korea; 4 Laboratory of Dendritic Cell Immunology, The Good Capital Institute for Immunology, Seoul, Republic of Korea

**Keywords:** CD32b, dendritic cells, GM-CSF, granulocyte-monocyte progenitor, *in vitro* differentiation, lineage heterogeneity, monocyte-dendritic cell progenitor, myeloid progenitors

The funder “a faculty research grant of Yonsei University College of Medicine to MC (6-2026-0018)” was erroneously omitted. The corrected **Funding** statement is given below:

“The author(s) declared that financial support was received for this work and/or its publication. This work was supported by grants from the National Research Foundation of Korea to CP (2019R1F1A1041700), HN (2021R1I1A1A01043872), and MC (2022R1A2C1091767); the Korea Health Technology R&D Project through the Korea Health Industry Development Institute to MC (HV22C0106); the Brain Korea 21 PLUS/FOUR Project for Medical Science, Yonsei University, a faculty research grant of Yonsei University College of Medicine to MC (6-2026-0018).”

The original version of this article has been updated.

