# Supplementary material for: CD32b defines distinct dendritic cell lineages generated from the culture of bone marrow with GM-CSF
Source: Front Immunol. 2026 May 29;17:1703978. doi: 10.3389/fimmu.2026.1703978 (PMC13259754; doi:10.3389/fimmu.2026.1703978)
Supplement: Supplementary Figure 1 — Time-course analysis of splenocyte cultures with GM-CSF and in vivo quantification of myeloid progenitor cell populations. (A) Representative flow cytogram and cell number of time-course culture of splenocytes with 1% GM-CSF-conditioned medium. Splenocytes were seeded at a density of 1×10^6^ cells in 24-well plate. Representative data are shown from three independent experiments in quadruplicate. (B) Number of total lives and myeloid progenitors (defined as Lineage(Lin)-CD117+) in BM and spleen. Lin includes Sca-1 and CD127 for excluding non-myeloid progenitors, i.e. common lymphoid progenitors (CLPs), multipotent progenitors (MPPs) and hematopoietic stem cells (HSCs). Data are shown from three independent experiments with biological triplicates using three individual mice. Error bars indicate mean ± SD. ns, not significant; *, p ≤ 0.05; **, p ≤ 0.01; ***, p ≤ 0.001; ****, p ≤ 0.0001. [file DataSheet1.pdf]

Supplementary Materials

Contents

Supplementary Figures	S1 - S18
Supplementary Tables	Table 1

Title
CD32b Defines Distinct Dendritic Cell Lineages Generated from The Culture of Bone Marrow with GM-CSF

Running Title
Progenitor-Driven Heterogeneity of GM-DCs

Author Names
Wanho Choi[1,2]†, Seul Hye Ryu,[1]†, Ji Soo Park[1,2], Da Eun Park[1,3], Min Kyung Chu[3]*, Hye Young Na[1,3]* and Chae Gyu Park[1,4]*

Author Affiliations
[1] Laboratory of Immunology, Severance Biomedical Science Institute, Yonsei University College of Medicine, Seoul, South Korea,
[2] Brain Korea 21 FOUR Project for Medical Science, Yonsei University College of Medicine, Seoul, South Korea,
[3] Department of Neurology, Severance Hospital, Yonsei University College of Medicine, Seoul, South Korea,
[4] The Good Capital Institute for Immunology, Seoul, South Korea

† These authors have contributed equally to this work
* These authors have contributed equally to this work

*** Correspondence:**

Chae Gyu Park, Ph.D.	Hye Young Na, Ph.D.	Min Kyung Chu, M.D., Ph.D.
ChaeGyu@goodcapital.org	HYEYNA@yuhs.ac	chumk@yonsei.ac.kr

Supp Figure 1

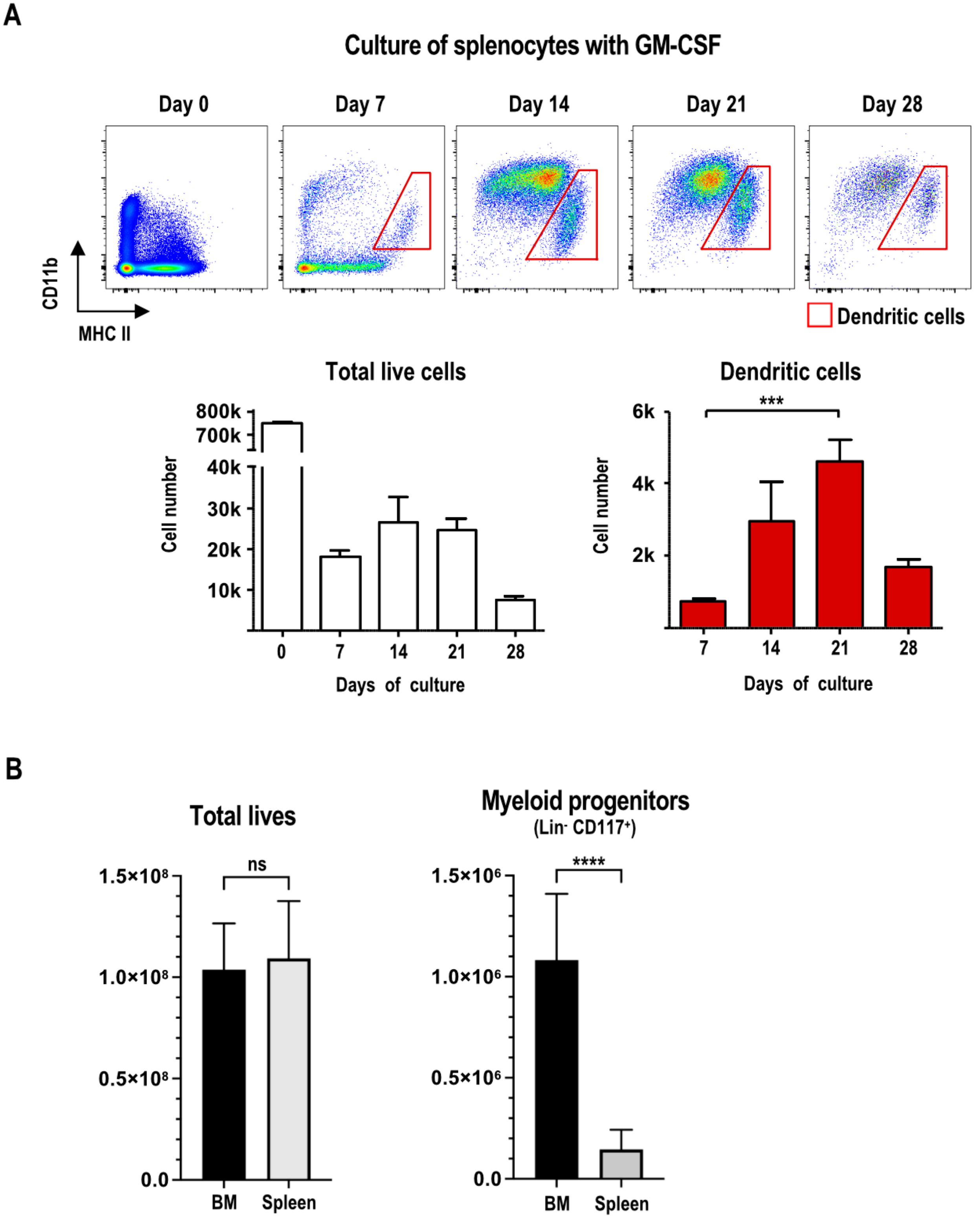

Supplementary Figure 1. **Time-course analysis of splenocyte cultures with GM-CSF and *in vivo* quantification of myeloid progenitor cell populations.**

(A) Representative flow cytogram and cell number of time-course culture of splenocytes with 1% GM-CSF-conditioned medium. Splenocytes were seeded at a density of 1×10^6 cells in 24-well plate. Representative data are shown from three independent experiments in quadruplicate.

(B) Number of total lives and myeloid progenitors (defined as Lineage(Lin)$^-$CD117$^+$) in BM and spleen. Lin includes Sca-1 and CD127 for excluding non-myeloid progenitors, i.e. common lymphoid progenitors (CLPs), multipotent progenitors (MPPs) and hematopoietic stem cells (HSCs). Data are shown from three independent experiments with biological triplicates using three individual mice. Error bars indicate mean ± SD. ns, not significant; *, $p \leq 0.05$; **, $p \leq 0.01$; ***, $p \leq 0.001$; ****, $p \leq 0.0001$.

Supp Figure 2

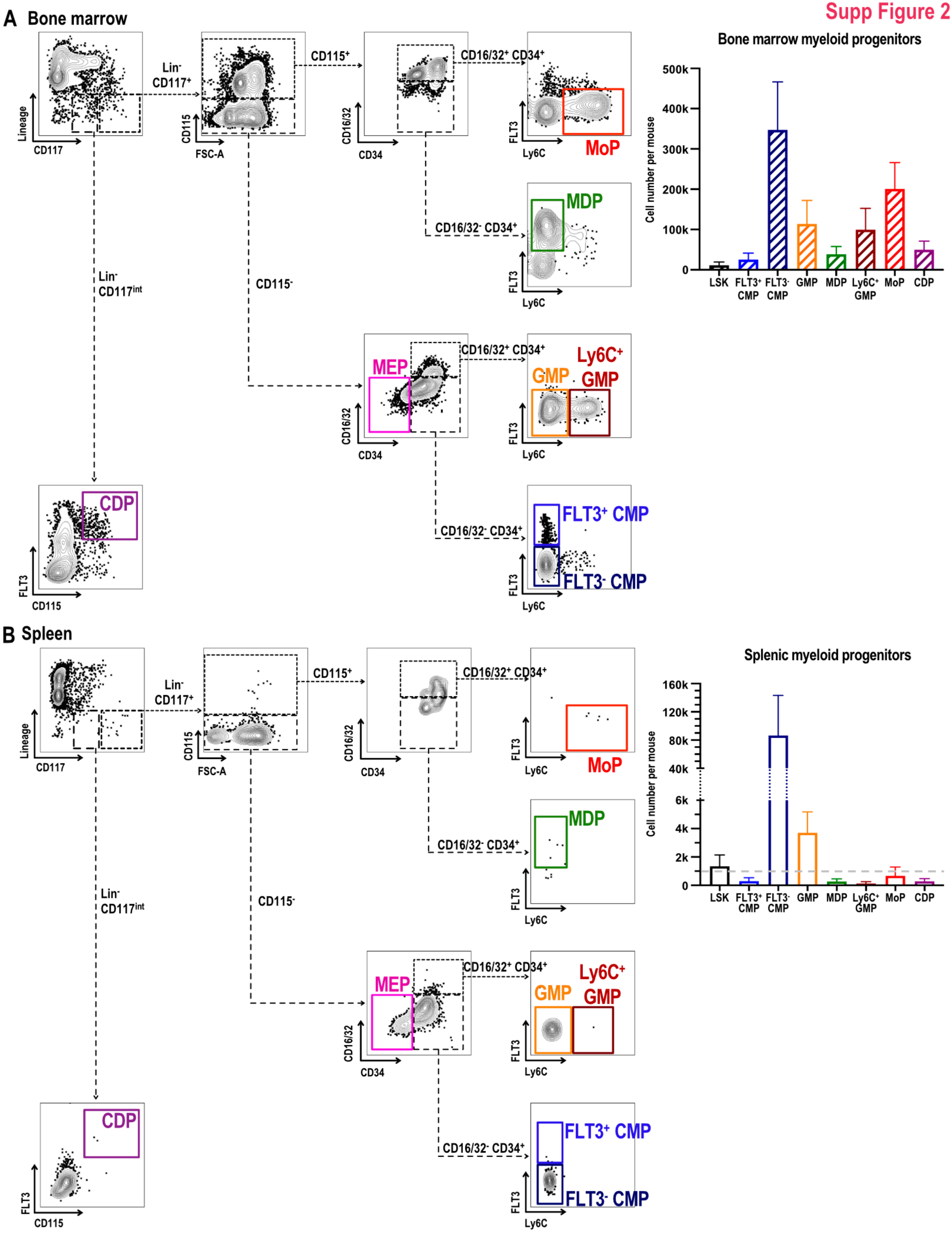

<u>Supplementary Figure 2</u>. Marker-based flow cytometric identification of myeloid progenitors in BM and spleen.

(A-B) Gating strategy and quantitative distribution of myeloid progenitors from (A) BM and (B) spleen by flow cytometry. Lin of myeloid progenitors includes Sca-1 and CD127. LSK (defined as $Lin^{-}Sca\text{-}1^{+}c\text{-}Kit^{+}$) includes HSC and MPP; $FLT3^{+}$ CMP; $FLT3^{+}$ Common Myeloid Progenitor defined as $Lin^{-}c\text{-}Kit^{+}CSF\text{-}1R^{-}CD16/32^{-}CD34^{+}Ly6C^{-}FLT3^{+}$, $FLT3^{-}$ CMP; $FLT3^{-}$ Common Myeloid Progenitor defined as $Lin^{-}c\text{-}Kit^{+}CSF\text{-}1R^{-}CD16/32^{-}CD34^{+}Ly6C^{-}FLT3^{-}$, GMP; Granulocyte Monocyte Progenitor defined as $Lin^{-}c\text{-}Kit^{+}CSF\text{-}1R^{-}CD16/32^{+}CD34^{+}Ly6C^{-}FLT3^{-}$, $Ly6C^{+}$ GMP; $Ly6C^{+}$ GMP defined as $Lin^{-}c\text{-}Kit^{+}CSF\text{-}1R^{-}CD16/32^{+}CD34^{+}Ly6C^{+}FLT3^{-}$, MEP; Megakary-ocyte Erythrocyte Progenitor defined as $Lin^{-}c\text{-}Kit^{+}$ $CSF\text{-}1R^{-}CD16/32^{-}CD34^{-}$, MDP;Macrophage Dendritic cell Progenitor defined as $Lin^{-}c\text{-}Kit^{+}CSF\text{-}1R^{+}CD16/32^{-}CD34^{+}Ly6C^{-}FLT3^{+}$, MoP; Mono-cyte Progenitor defined as $Lin^{-}c\text{-}Kit^{+}CSF\text{-}1R^{+}CD16/32^{+}CD34^{+}$ $Ly6C^{+}FLT3^{-}$, CDP; Common Dendritic cell Progenitor defined as $Lin^{-}c\text{-}Kit^{int}CSF\text{-}1R^{+}FLT3^{+}$. Representative data are shown from five biologically independent samples (n=5).

Supp Figure 3

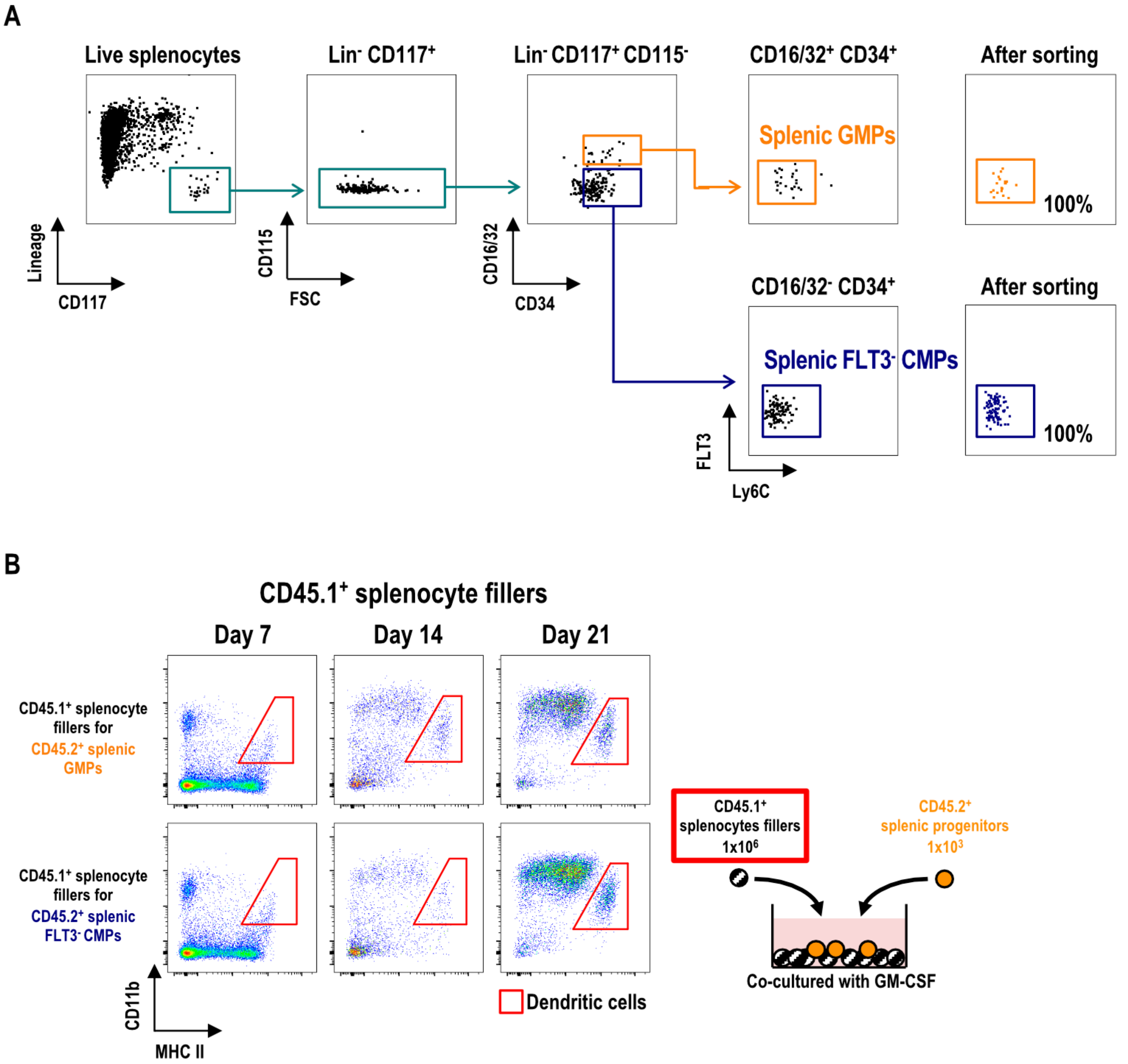

Supplementary Figure 3. **Gating strategy of splenic myeloid progenitors and representative flow cytograms of CD45.1$^+$ splenocyte fillers.**
(A) Gating strategy and cell purity of sorted splenic GMPs and FLT3$^-$ CMPs.
(B) Representative flow cytograms of splenocyte fillers in **Fig.1, A and B**. CD45.1$^+$ splenocyte fillers were seeded at a density of 1×10^6 cells in 24-well plate.

Supp Figure 4

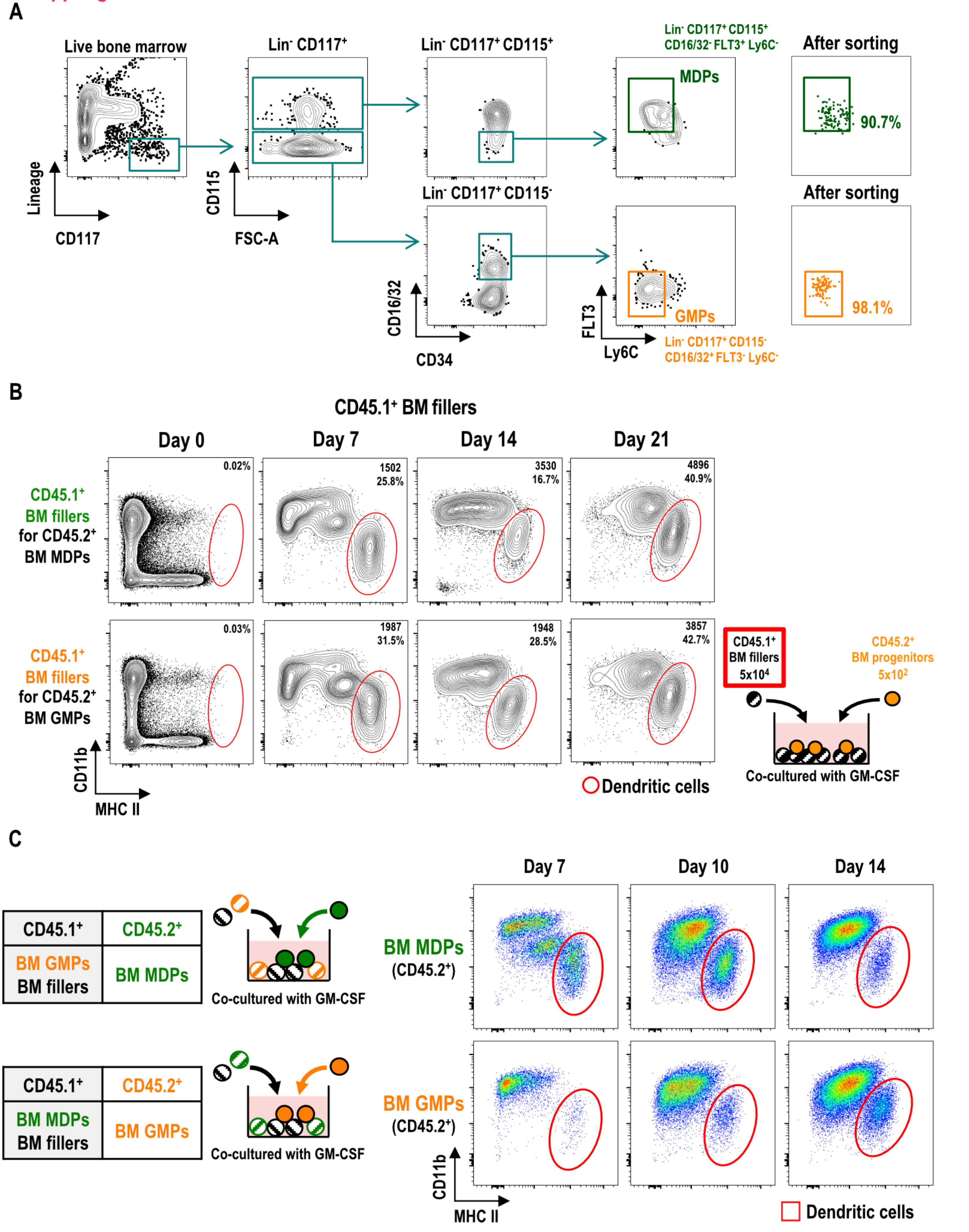

<u>Supplementary Figure 4</u>. Co-culture conditions of CD45.1$^+$ BM fillers and gating strategy for BM GMPs and MDPs.

(A) Gating strategy and cell purity of sorted BM GMPs and MDPs.

(B) Representative flow cytograms of BM fillers in **Fig.2 A**. CD45.1$^+$ BM fillers were seeded at a density of 5×10^4 cells and CD45.2$^+$ sorted BM progenitor cells were seeded at a density of 5×10^2 cells in 48-well plate.

(C) Graphical scheme and representative flow cytograms of simultaneous co-culture experiment of BM fillers, BM MDPs and GMPs. CD45.2$^+$ and CD45.1$^+$ sorted progenitors were co-seeded at a density of 5×10^2 cells, together with 5×10^4 CD45.1$^+$ BM filler cells, in 48-well plates.

Supp Figure 5

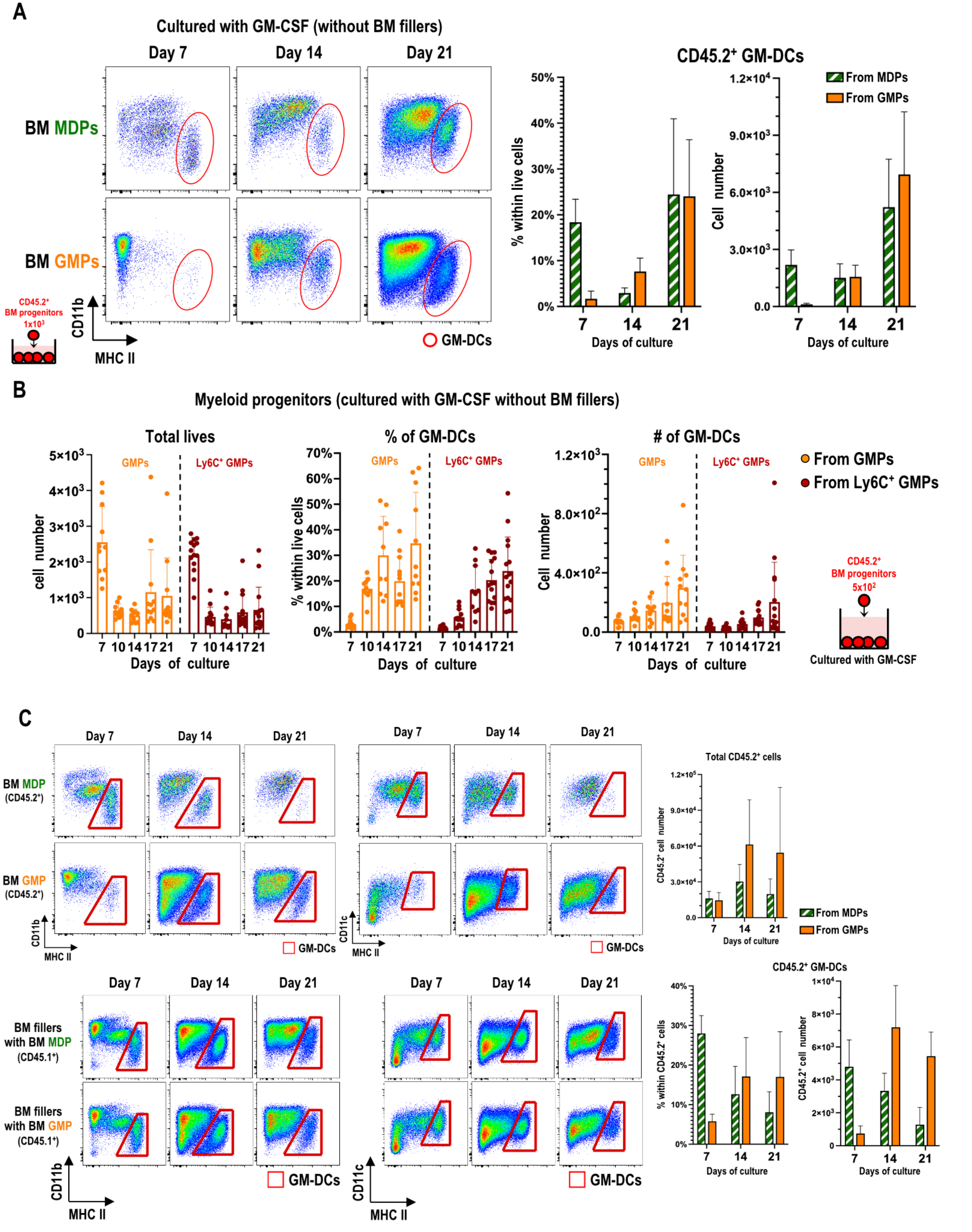

Supplementary Figure 5. Distinct progenitor subsets follow different GM-DC differentiation kinetics from culture without BM fillers.

(A) Representative flow cytogram and cell number of time-course culture of BM GMPs and BM MDPs without BM fillers under 5% GM-CSF-conditioned medium. Sorted cells were seeded at a density of 1×10^3 cells in 24-well plate. Representative data are shown from 3 independent experiments in triplicate.

(B) Cell number and proportion of time-course culture of BM GMPs and $Ly6C^+$ GMPs without BM fillers under 5% GM-CSF-conditioned medium. Sorted cells were seeded at a density of 5×10^2 cells in 48-well plate. Representative data are shown from 3 independent experiments in quadruplicate.

(C) Representative flow cytogram and cell number of time-course culture of BM GMPs and BM MDPs with twofold higher cell numbers. Sorted $CD45.2^+$ progenitor cells were seeded at a density of 1×10^3 cells and BM filler cells were seeded at a density of $1x10^5$ cells in 24-well plate. Representative data are shown from 3 independent experiments in triplicate. Error bars indicate mean ± SD across multi-plicate samples.

Supp Figure 6

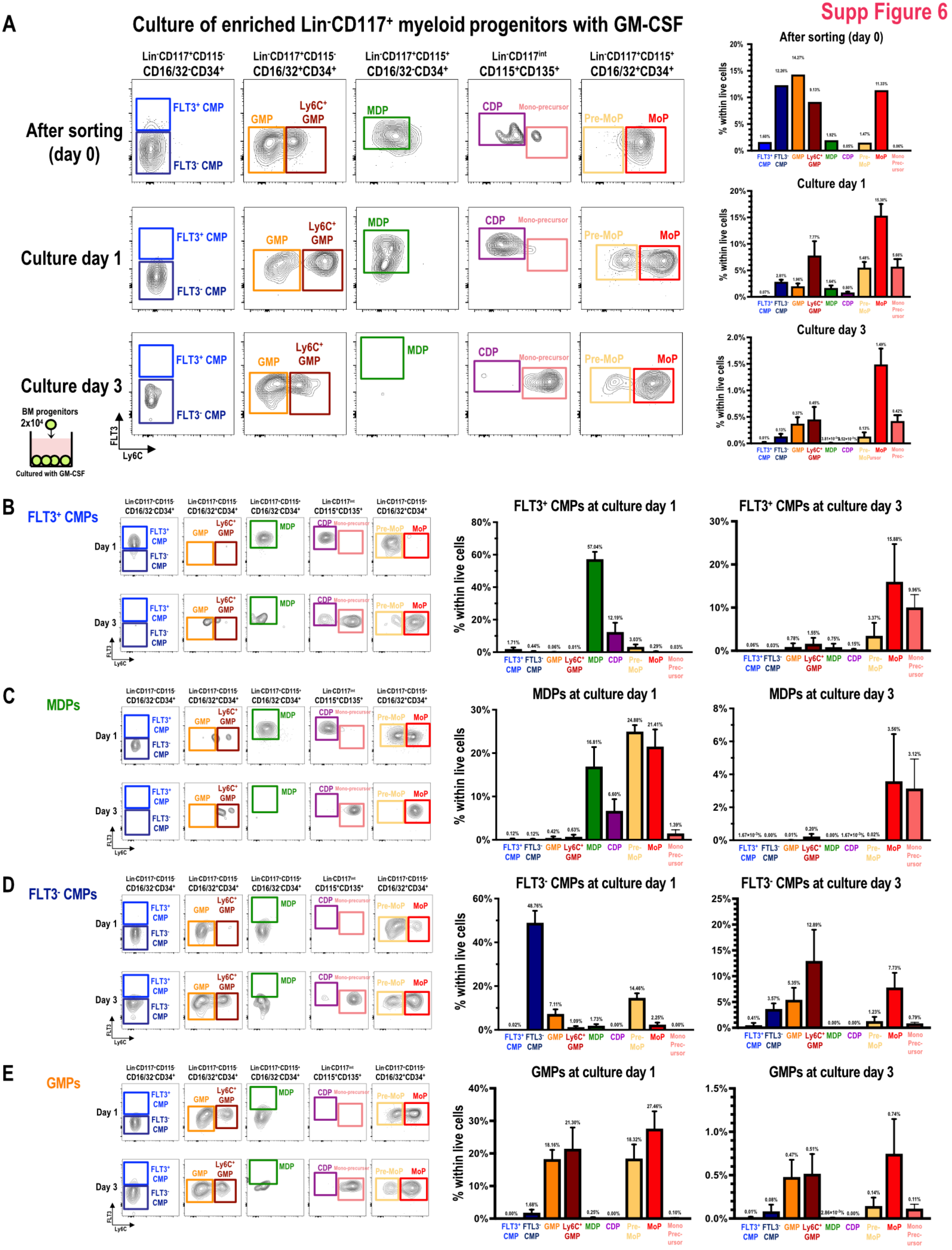

Supplementary Figure 6. Flow cytometric analysis of BM myeloid progenitors under short-term GM-CSF culture conditions.

(A) Representative flow cytograms and quantification of myeloid progenitors from $Lin^{-}Sca\text{-}1^{-}CD127^{-}CD117^{+}$ enriched BM cells cultured with 3% GM-CSF-conditioned medium for 0, 1 or 3 days. Enriched cells were seeded at a density of 2×10^{4} cells and cultured for 1 or 3 days in 24-well plates.

(B–E) Representative flow cytograms and bar graphs showing the differentiation outcomes of sorted (B) $FLT3^{+}$ CMPs, (C) $FLT3^{-}$ CMPs, (D) MDPs, and (E) GMPs after 1 or 3 days of culture with GM-CSF. Each progenitor population was seeded at a density of 3×10^{3}-5×10^{3} cells and cultured for 1 or 3 days in 48-well plates. Data shown from three independent experiments performed in duplicate. Error bars indicate mean ± SD.

Supp Figure 7

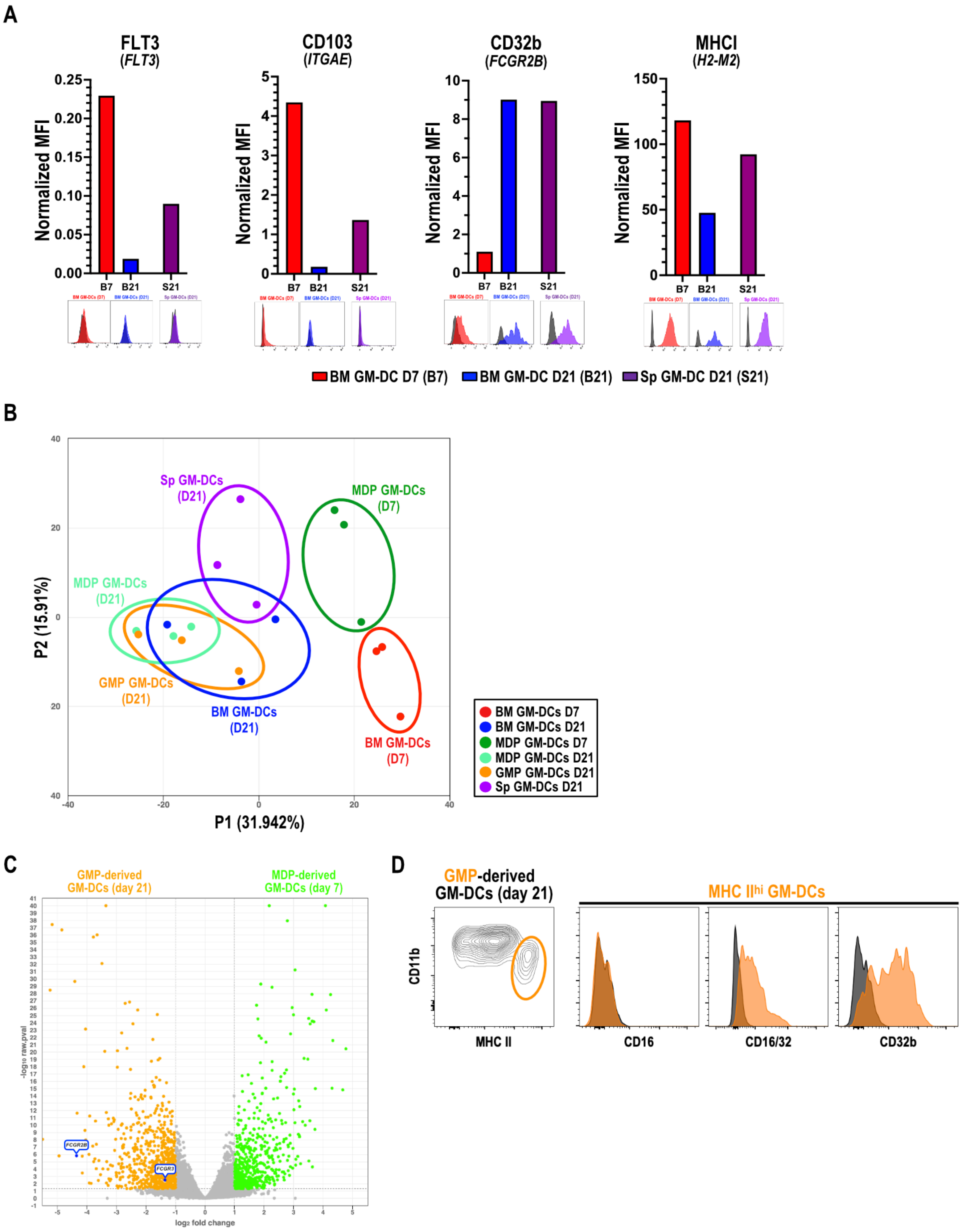

Supplementary Figure 7. Transcriptomic and phenotypic characterization of developmentally distinct MDP- and GMP-derived GM-DC subsets.

(A) Flow cytometric validation of selected surface markers identified in DEG analysis. Normalized Mean fluorescence index (MFI) values of four representative markers from each subset are shown. Normalized MFI was calculated by subtracting the isotype control mean fluorescence from the sample mean fluorescence, and then dividing the result by the isotype control mean. Representative flow cytometric histograms are shown with isotype controls overlaid in gray. Representative data are shown from two independent experiments.

(B) Principal component analysis (PCA) of bulk RNA-seq data from GM-DCs sorted at day 7 and day 21. DCs were collected from splenocyte cultures (day 21), BM cultures (day 7 and 21) and from cultures of sorted MDPs (day 7 and day 21) or GMPs (day 21) under GM-CSF conditions.

(C) Volcano plot of bulk RNA-seq data comparing MDP-derived GM-DCs (day 7) and GMP-derived GM-DCs (day 21). Selected genes (*FCGR2B*, *FCGR3*) are highlighted with blue dot.

(D) Representative Flow cytograms of GMP-derived cells cultured with GM-CSF. Overlaid black histograms represent the fluorescence-minus-one (FMO) controls.

Supp Figure 8

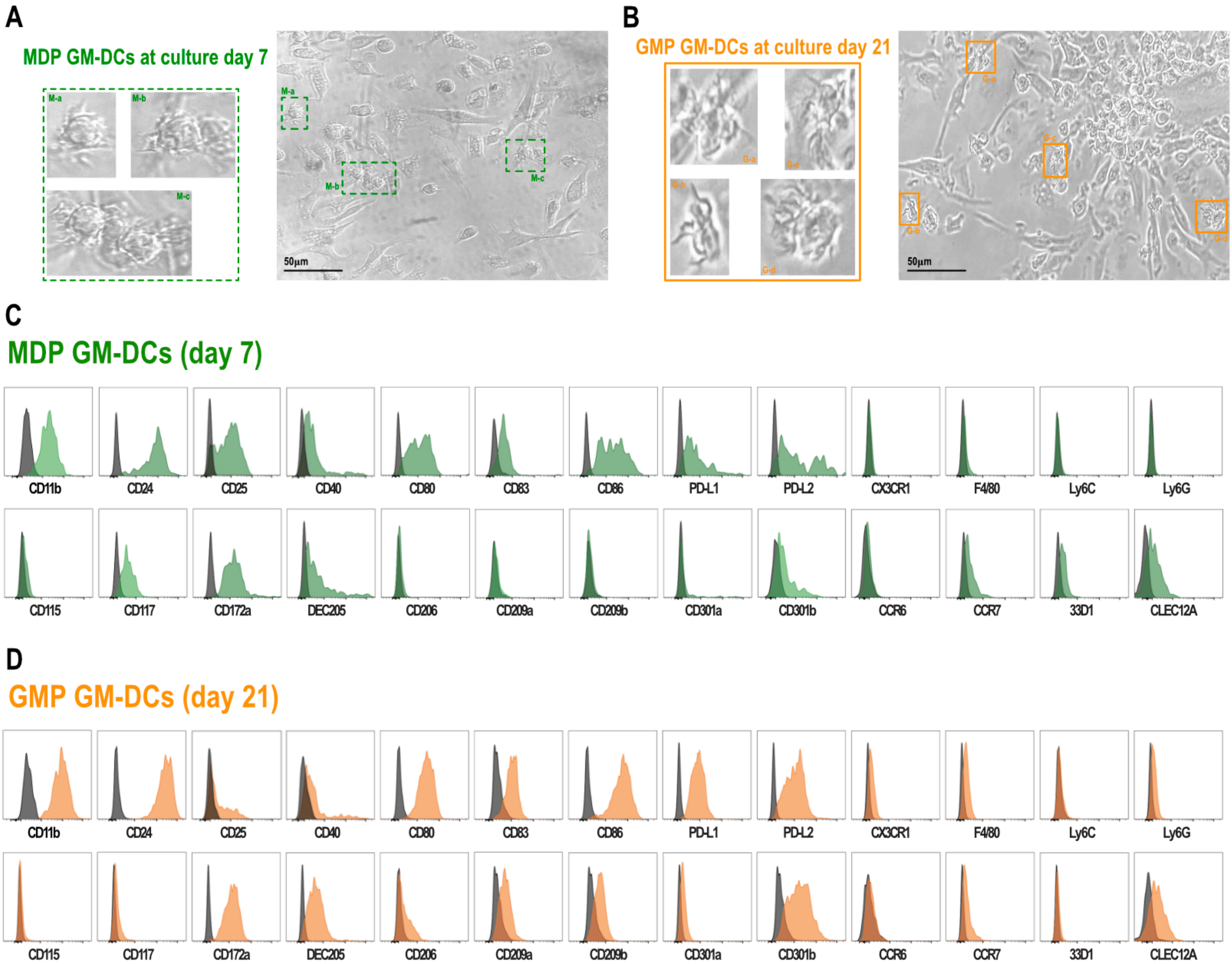

Supplementary Figure 8. Morphology and surface marker expression of MDP GM-DCs (day 7) and GMP GM-DCs (day 21).

(A–B) Representative phase-contrast micrographs showing the morphology of (A) MDP GM-DCs at day 7 and (B) GMP GM-DCs at day 21 of culture. Images were captured at 200x magnification. Scale bars: 50 µm.

(C–D) Flow cytometric histograms of various myeloid markers. Representative flow cytometric histograms are shown with isotype controls overlaid in gray. Representative data are shown from two independent experiments.

Supp Figure 9

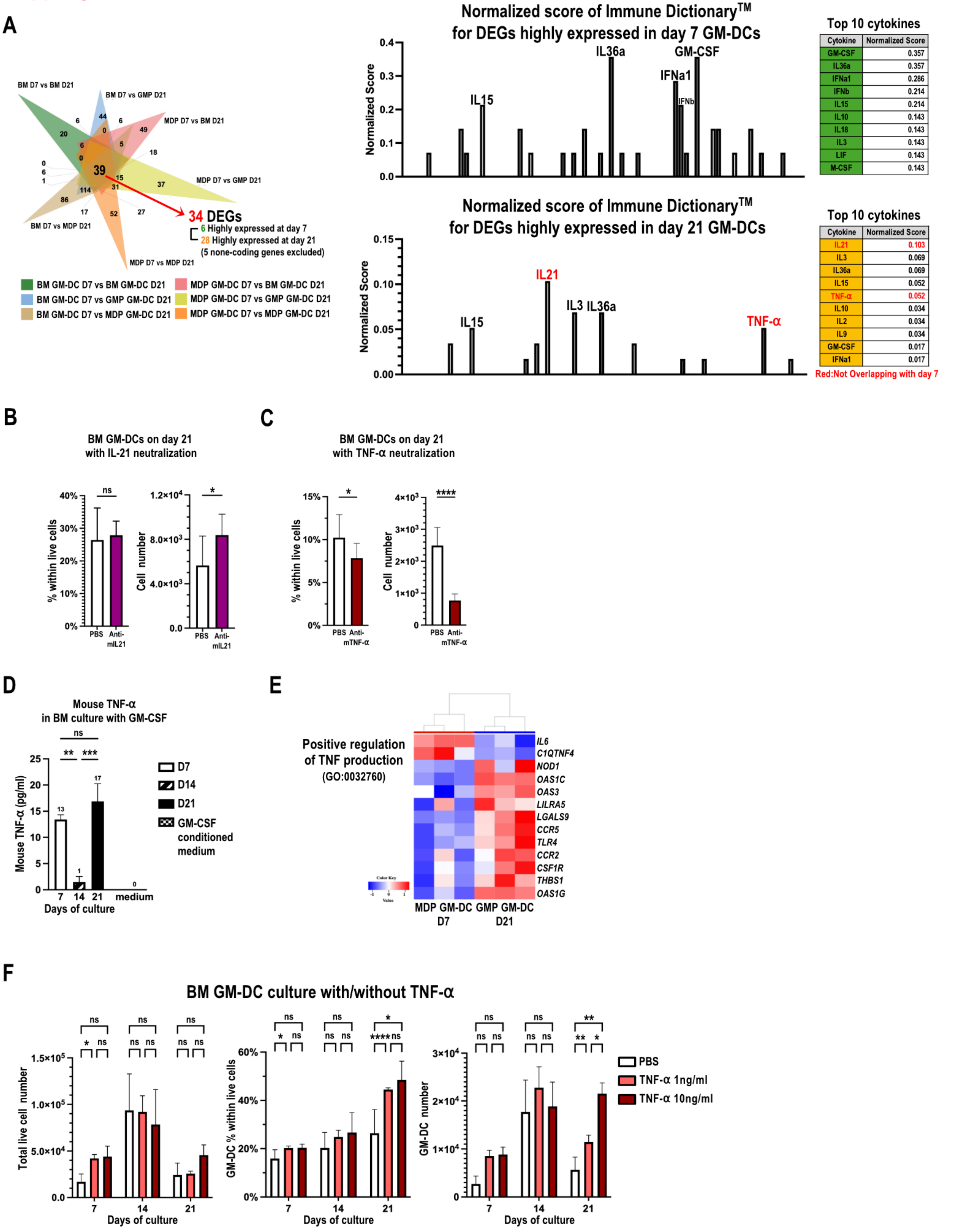

Top 10 cytokines

Cytokine	Normalized Score
GM-CSF	0.357
IL36a	0.357
IFNa1	0.286
IFNb	0.214
IL15	0.214
IL10	0.143
IL18	0.143
IL3	0.143
LIF	0.143
M-CSF	0.143

Top 10 cytokines

Cytokine	Normalized Score
IL21	0.103
IL3	0.069
IL36a	0.069
IL15	0.052
TNF-α	0.052
IL10	0.034
IL2	0.034
IL9	0.034
GM-CSF	0.017
IFNa1	0.017

Supplementary Figure 9. TNF-α promotes the differentiation of CD32b$^+$ GM-DCs.

(A) Bioinformatic prediction of key cytokines involved in GM-DC differentiation. Genes significantly upregulated in GM-DCs on day 7 versus day 21 (fold change > 4, $p < 0.01$) were used as input for a cytokine prediction analysis (Immune Dictionary™). Cytokines were ranked based on a normalized score. Normalized score derived from categorical binning of gene-level expression (0 = low, 1 = intermediate, 2 = high).

(B) Effect of IL-21 neutralization on GM-DC generation. BM cells (1×10^5) were cultured with 3% GM-CSF-conditioned medium and treated with either PBS or an anti-mouse IL-21 antibody (10 μg/mL). The frequency and number of DCs were assessed on day 21.

(C) Effect of TNF-α neutralization on GM-DC generation. BM cells (5×10^4 cells/well) were cultured with 3% GM-CSF-conditioned medium and treated with either PBS or an anti-mouse TNF-α antibody (10 μg/mL). The frequency and number of DCs were analyzed on day 21.

(D) Quantification of TNF-α in culture supernatants. Supernatants from BM cultures with 3% GM-CSF-conditioned medium (1×10^5 cells/well) were harvested on days 7, 14, and 21 and analyzed by ELISA. Medium containing only GM-CSF served as the control. Data are from two independent experiments.

(E) Heatmap of genes associated with the Gene Ontology (GO) term "Positive regulation of TNF production" (GO:0032760), comparing day 7 MDP-derived GM-DCs with day 21 GMP-derived GM-DCs.

(F) Effect of exogenous TNF-α on BM cultures. BM cells (5×10^4 cells/well) were cultured with 3% GM-CSF-conditioned medium and treated with the indicated concentrations of recombinant mouse TNF-α. Total cell number, DC frequency, and absolute DC number were quantified on days 7, 14, and 21. Data are representative of two independent experiments performed in triplicate. Statistical significance was determined using a two-way ANOVA followed by Tukey's multiple comparison test. Error bars represent mean ± SD. ns, not significant; *, $p \leq 0.05$; **, $p \leq 0.01$; ***, $p \leq 0.001$; ****, $p \leq 0.0001$.

Supp Figure 10

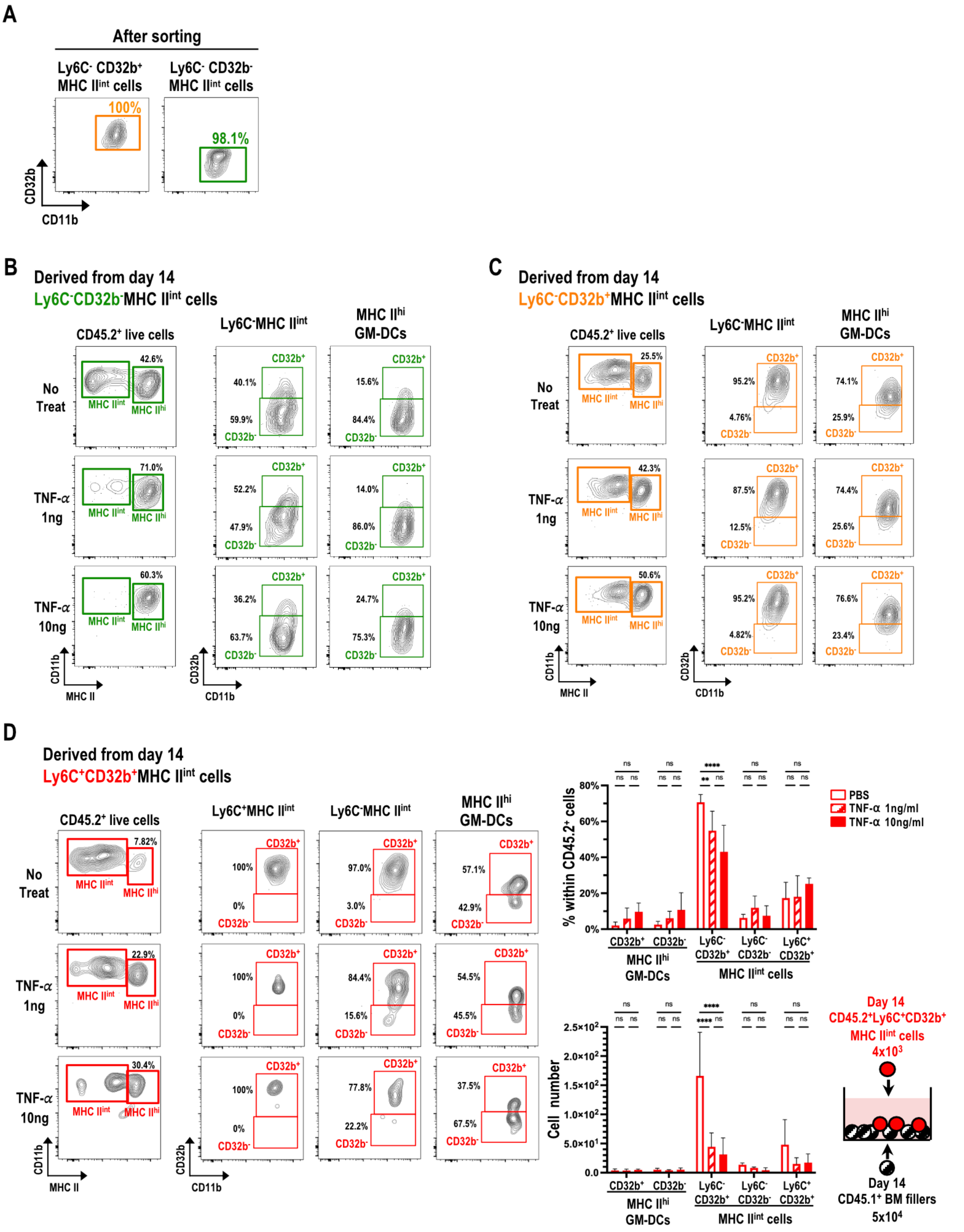

Supplementary Figure 10. Sorting purity and co-culture of CD32b^{+} and CD32b^{-} MHC II^{int} subsets from BM culture with GM-CSF.

(A) Sorting purity of pre-DC candidates. CD32b^{+} pre-GM-DC (MHC II^{int}Ly6C^{-}CD32b^{+}) and CD32b^{-} pre-GM-DC (MHC II^{int}Ly6C^{-}CD32b^{-}) populations are sorted from 14-day cultured BM with 3% GM-CSF-conditioned medium.

(B-C) Representative flow cytograms of co-cultured (B) CD32b^{+} and (C) CD32b^{-} MHC II^{int} populations with 14-day cultured CD45.1^{+} BM fillers and GM-CSF.

(D) Representative flow cytograms and quantification of co-cultured Ly6C^{+}CD32b^{+} populations with 14-day cultured CD45.1^{+} BM fillers and GM-CSF. Representative flow cytograms are shown. Data are shown from two independent experiments in triplicate. Statistical significance was determined using a two-way ANOVA followed by Tukey's multiple comparison test. Error bars indicate mean ± SD across triplicate samples. *, $p \leq 0.05$; **, $p \leq 0.01$; ***, $p \leq 0.001$; ****, $p \leq 0.0001$.

Supp Figure 11

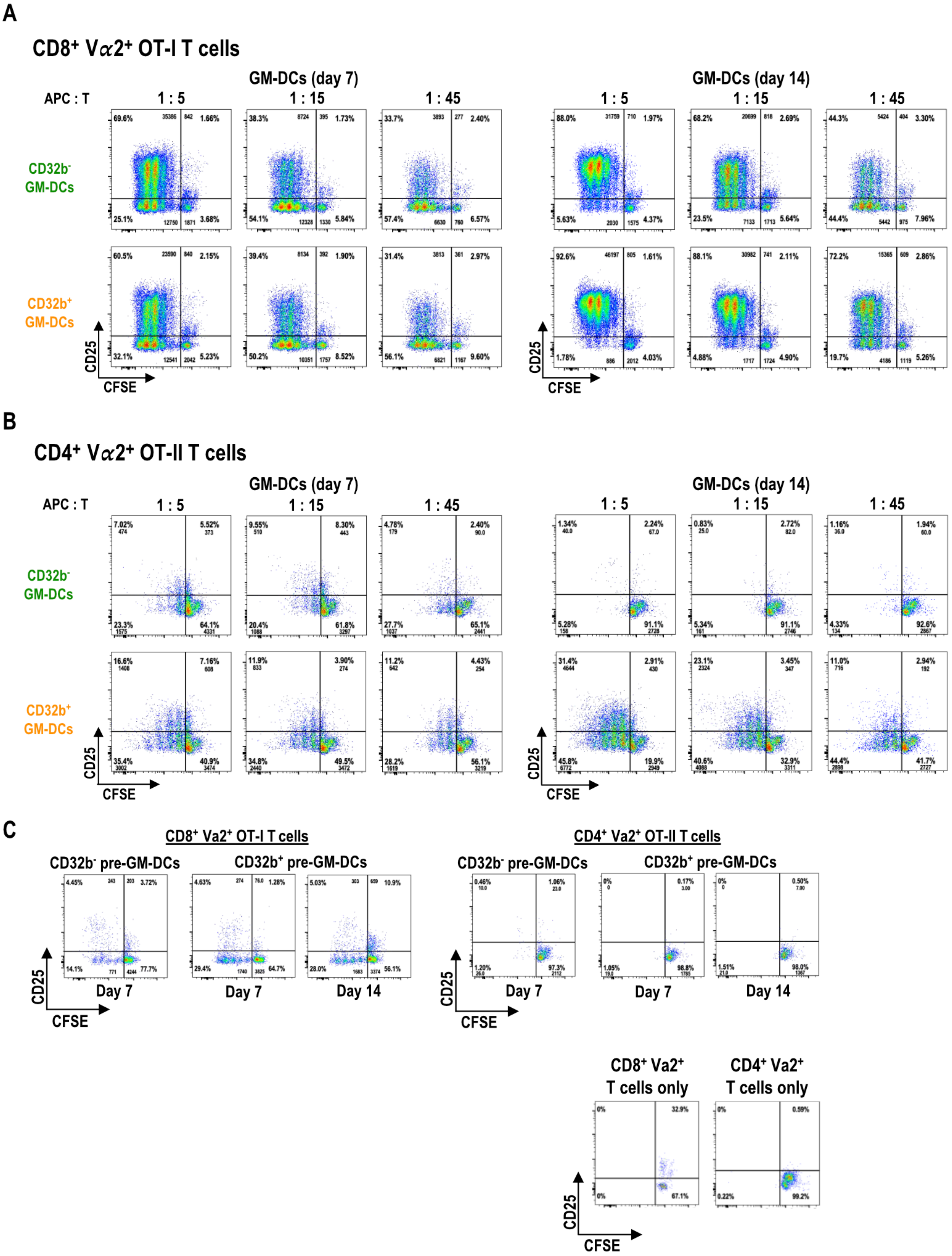

<u>Supplementary Figure 11</u>. Representative flow cytograms of antigen presentation by GM-DCs in Figure 6.

(A–B) Representative flow cytograms of (A) OT-I ($CD8^+$) and (B) OT-II ($CD4^+$) T cell proliferation in response to $CD32b^+$ or $CD32b^-$ GM-DCs sorted at day 7 or 14.

(C) Representative flow cytograms of T cell priming assay using pre-GM-DC populations and T cells only. $Ly6C^-CD32b^-$ and $Ly6C^-CD32b^+$ pre-GM-DCs were co-cultured with OT-I ($CD8^+$) and OT-II ($CD4^+$) T cells at a 1:5 ratio.

Supp Figure 12

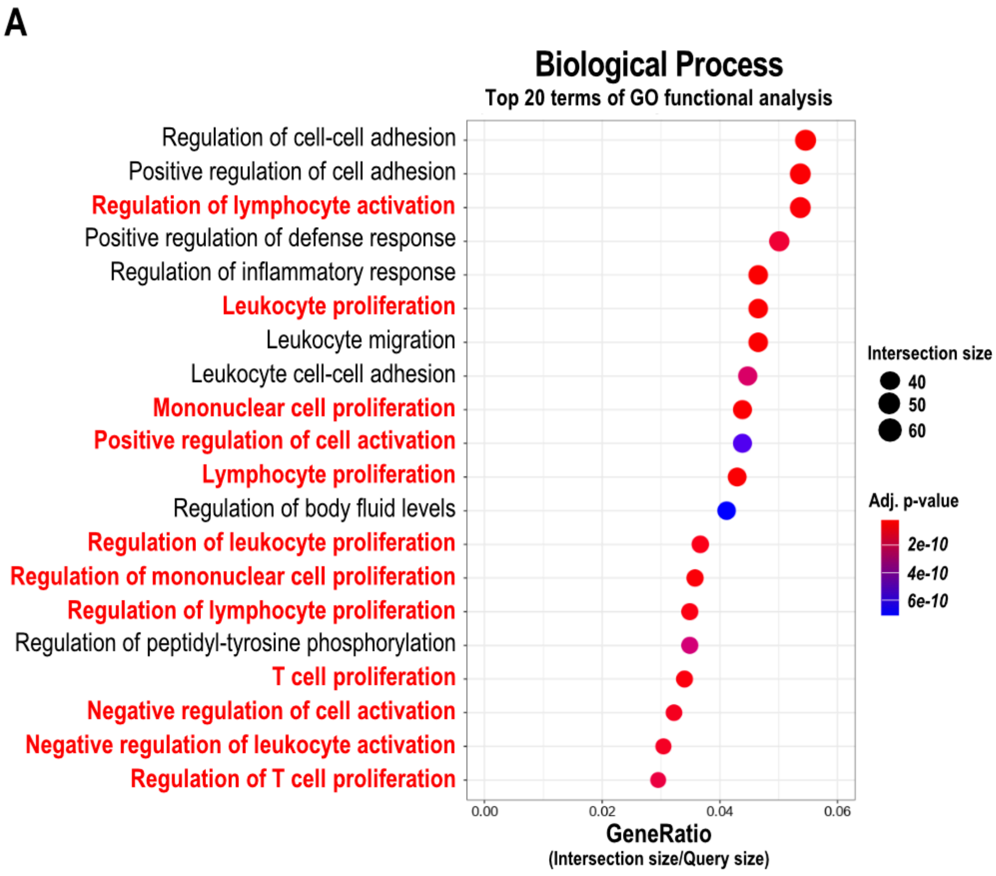

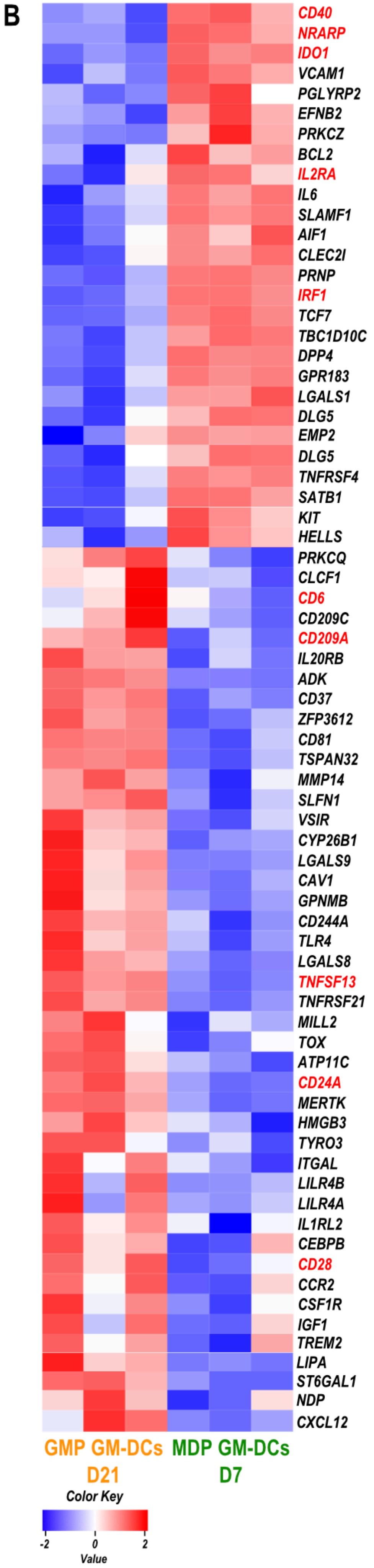

__Supplementary Figure 12__. **Transcriptional and functional differences between GM-DCs on day 7 or day 21 revealed by GO analysis.**

(A) Gene ontology:Biological process enrichment analysis (GO:BP) from differentially expressed genes between MDP GM-DCs (day 7) and GMP GM-DCs (day 21). Top 20 enriched terms are shown after size-based filtering.

(B) Heatmap of genes associated with the activation and proliferation-associated GO terms from (A).

Supp Figure 13

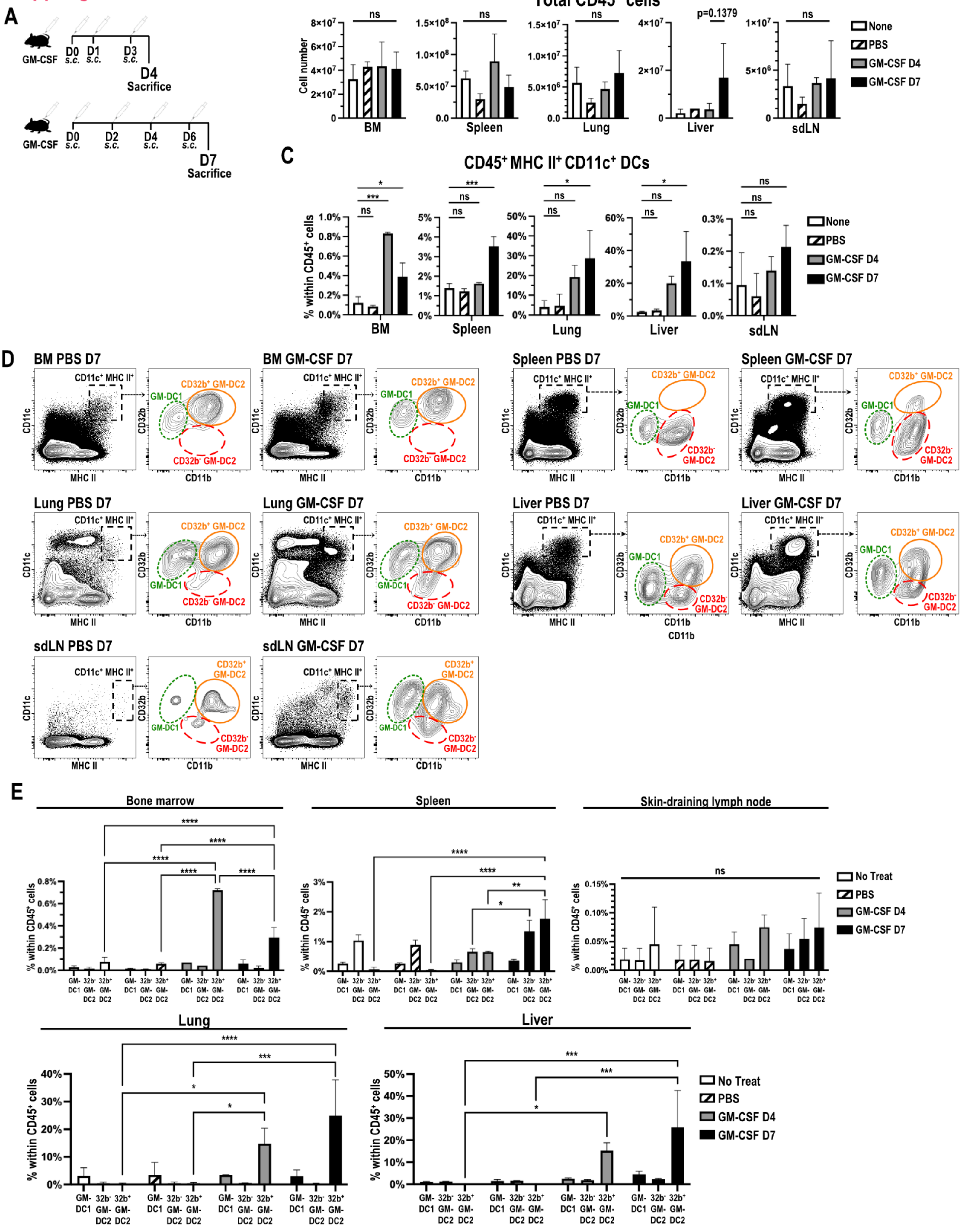

Supplementary Figure 13. Organ-specific kinetics of DC subset generation following *in vivo* GM-CSF administration.

(A) Scheme of the experimental design. Mice were subcutaneously injected with recombinant mouse GM-CSF, and organs were harvested on day 4 or day 7 for flow cytometric analysis.

(B) Total number of $CD45^+$ cells in the BM, spleen, lung, liver, and skin-draining lymph nodes (sdLNs) on day 4 and day 7 post-injection.

(C) Percentage of MHC II^+CD11c^+ DC populations within $CD45^+$ cells in the BM, spleen, lung, liver, and sdLNs on day 4 and day 7 post-injection.

(D) Representative flow cytogram of MHC II^+CD11c^+ DC subsets in various organs on day 7. MHC II^+CD11c^+ DC subsets were classified as $CD11b^-$ GM-DC1s, $CD11b^+CD32b^-$ GM-DC2s, and $CD11b^+CD32b^+$ GM-DC2s.

(E) DC subsets within the indicated organs on day 4 and day 7. Data are representative of two independent experiments with two biological replicates per group. Statistical significance was determined using a two-way ANOVA followed by Tukey's multiple comparison test. Error bars represent mean ± SD. ns, not significant; *, $p \leq 0.05$; **, $p \leq 0.01$; ***, $p \leq 0.001$; ****, $p \leq 0.0001$.

Supp Figure 14

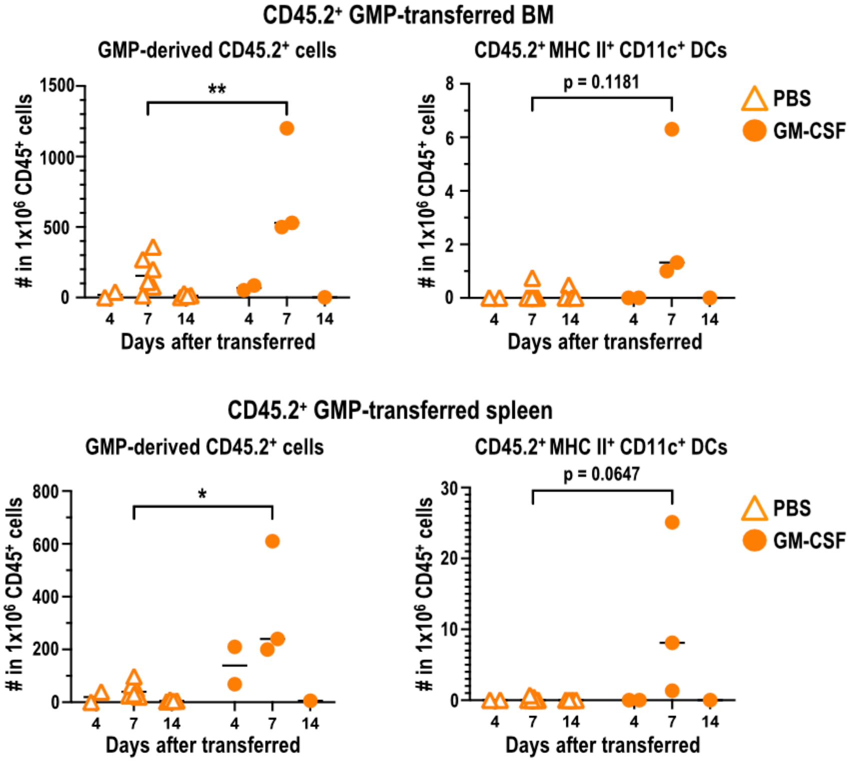

<u>Supplementary Figure 14.</u> Kinetics of adoptively transferred GMP-derived DC generation in BM and spleen following GM-CSF treatment. Cell number of $CD45.2^+$ GMP-derived cell and MHC II^+CD11c^+ DC counts in the BM and spleen following GM-CSF administration at day 4, 7, or 14.

Supp Figure 15

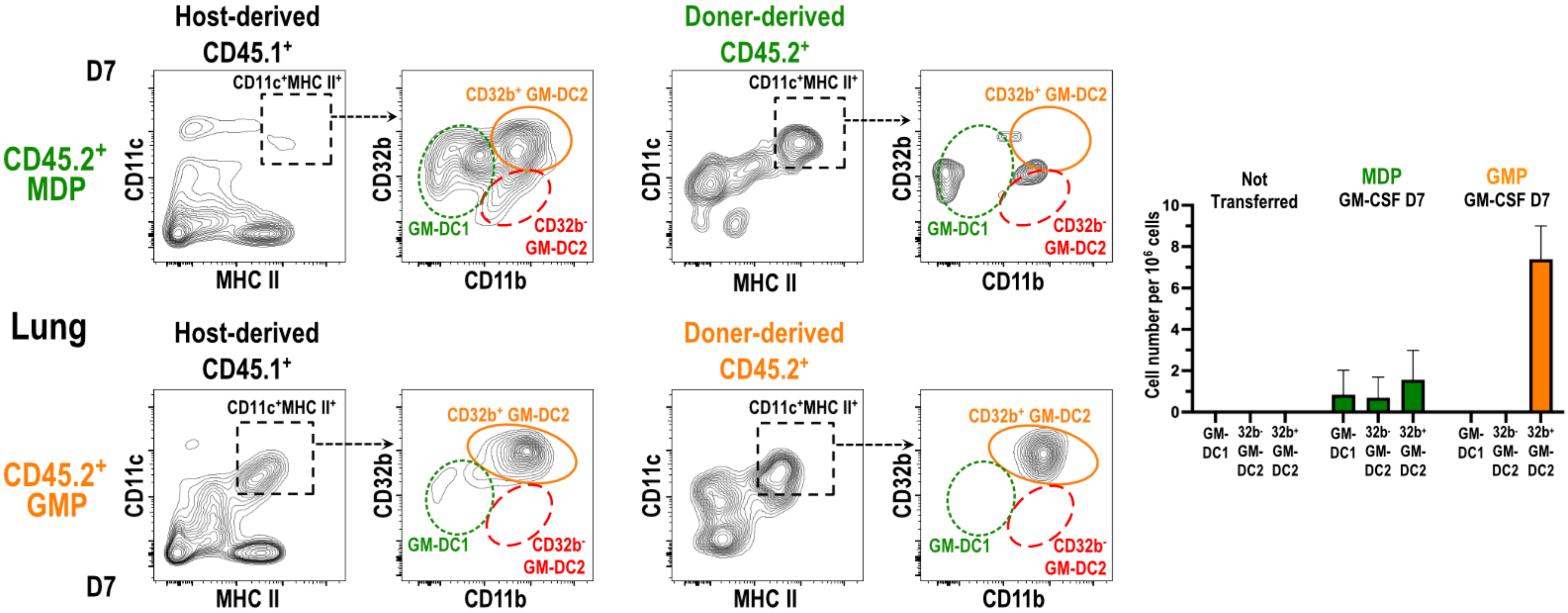

Supplementary Figure 15. Adoptively transferred GMP and MDP in lung at day 7 following GM-CSF treatment.

Representative flow cytograms and quantification of donor-derived lung DC subsets at day 7 following PBS or GM-CSF treatment. DCs were defined as MHC $II^{+}CD11c^{+}$ cells; $CD11b^{-}$ cells as GM-DC1s, and $CD11b^{+}$ GM-DC2s were further subdivided into $CD32b^{-}$ and $CD32b^{+}$ GM-DC2s. Donor cells were identified by $CD45.2^{+}$ gating. Representative flow cytograms are shown. Error bars indicate mean ± SD across duplicate biological replicates. Data are representative of two independent experiments.

Supp Figure 16

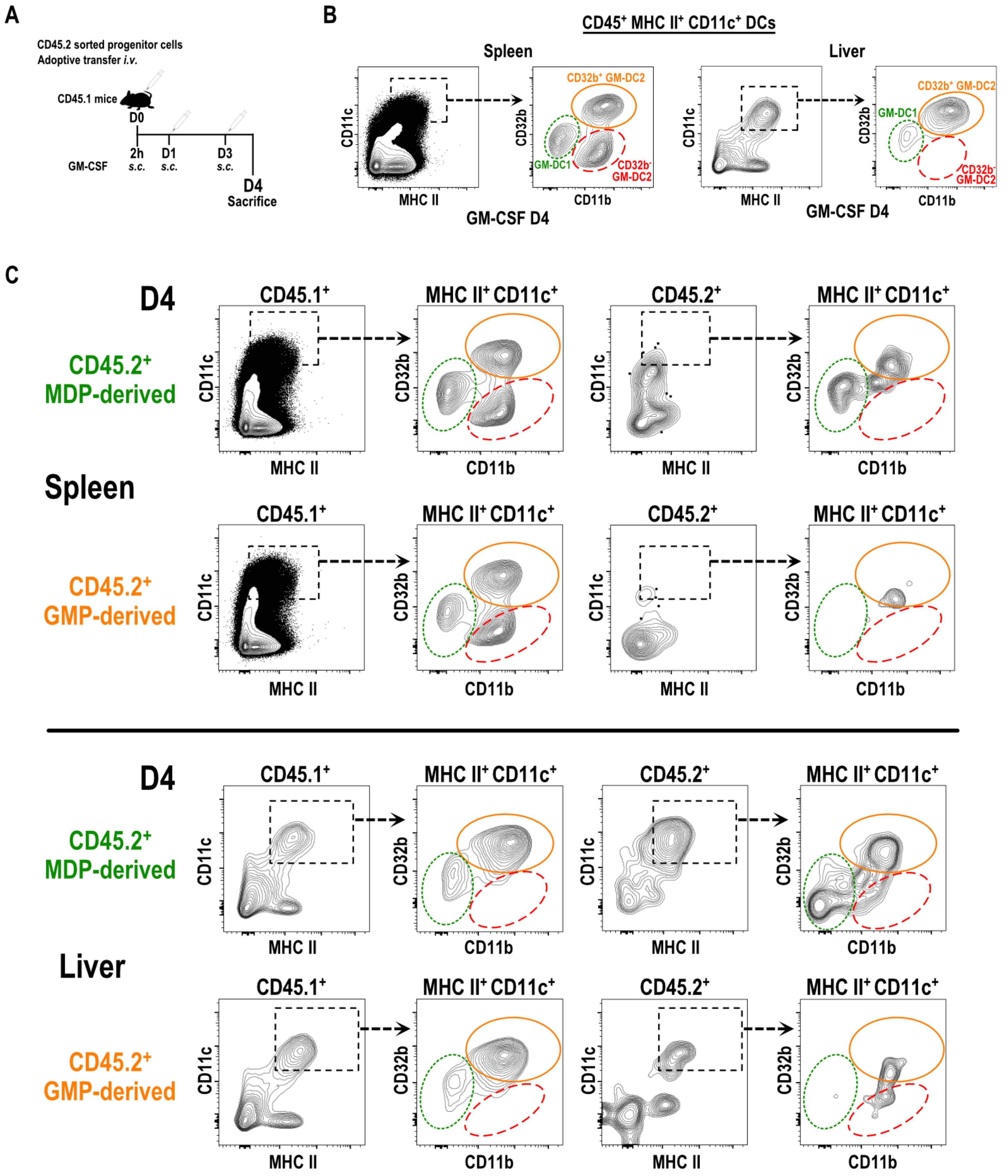

Supplementary Figure 16. Adoptively transferred GMP and MDP in spleen and liver at day 4 following GM-CSF treatment.

(A) Scheme of the *in vivo* adoptive transfer experiment. Sorted $CD45.2^+$ GMPs or MDPs were transferred into $CD45.1^+$ recipient mice, which were then treated with GM-CSF. Organs were harvested at day 4 for flow cytometric analysis.

(B) Representative flow cytograms of splenic and hepatic MHC II^+CD11c^+ DC subsets at day 4 following GM-CSF administration. Representative flow cytograms are shown. Data are representative of two independent experiments with two biological replicates per group.

(C) Representative flow cytograms of donor-derived splenic and hepatic GM-DC subsets on day 4. Donor cells were identified by $CD45.2^+$ gating. Representative flow cytograms are shown. Data are representative of two independent experiments with two biological replicates per group. Error bars represent mean ± SD.

Supp Figure 17

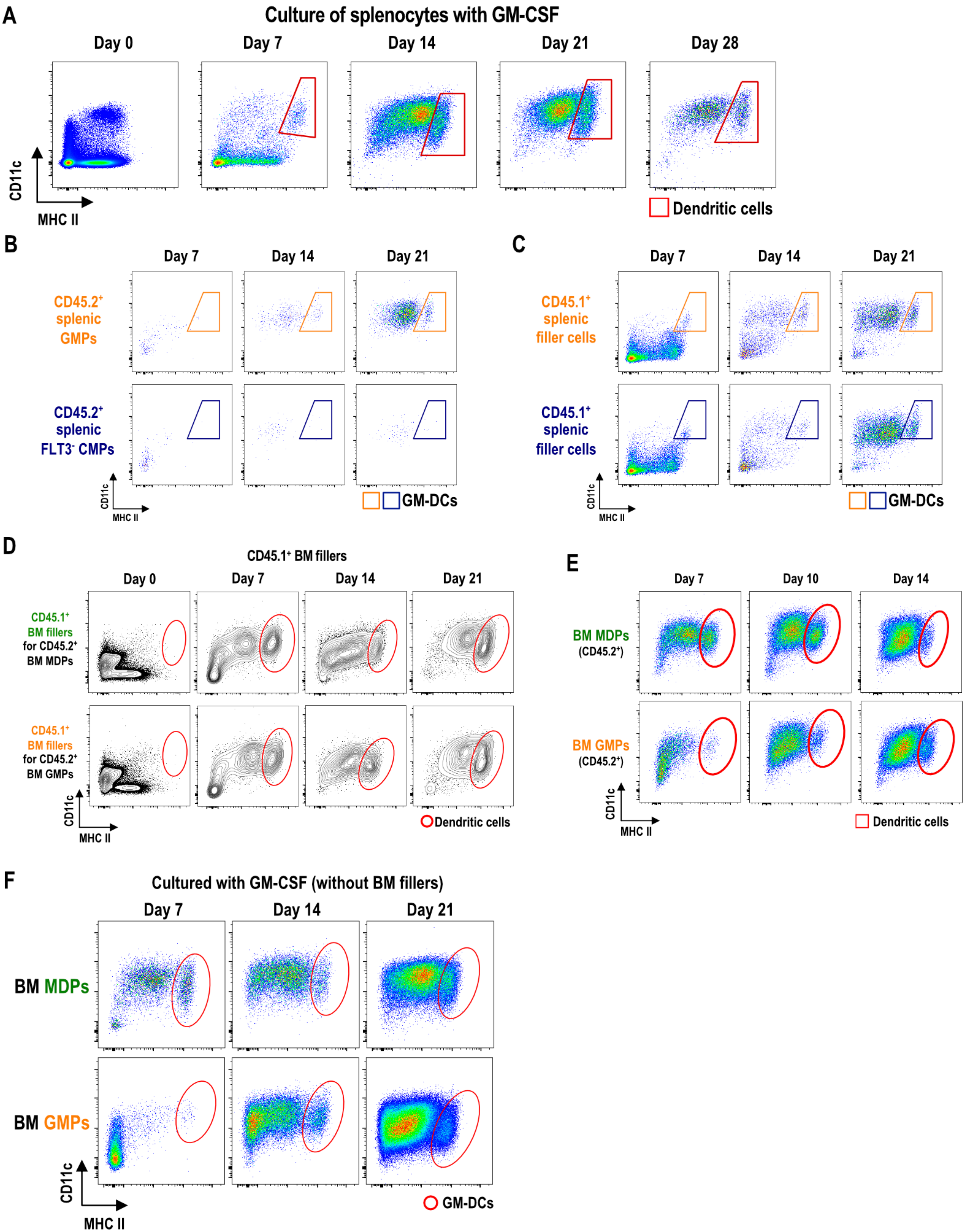

<u>Supplementary Figure 17.</u> Validation of GM-DC identification using MHC II and CD11c markers.

(A) Corresponding flow cytograms to **Figure S1 A**, showing MHC II versus CD11c expression.

(B) Corresponding flow cytograms of **Figure 1 A**, showing MHC II versus CD11c expression.

(C) Corresponding flow cytograms of **Figure S3 B**, showing MHC II versus CD11c expression.

(D) Corresponding flow cytograms of **Figure S4 B**, showing MHC II versus CD11c expression.

(E) Corresponding flow cytograms of **Figure S4 C**, showing MHC II versus CD11c expression.

(F) Corresponding flow cytograms of **Figure S5 A**, showing MHC II versus CD11c expression.

Supp Figure 18

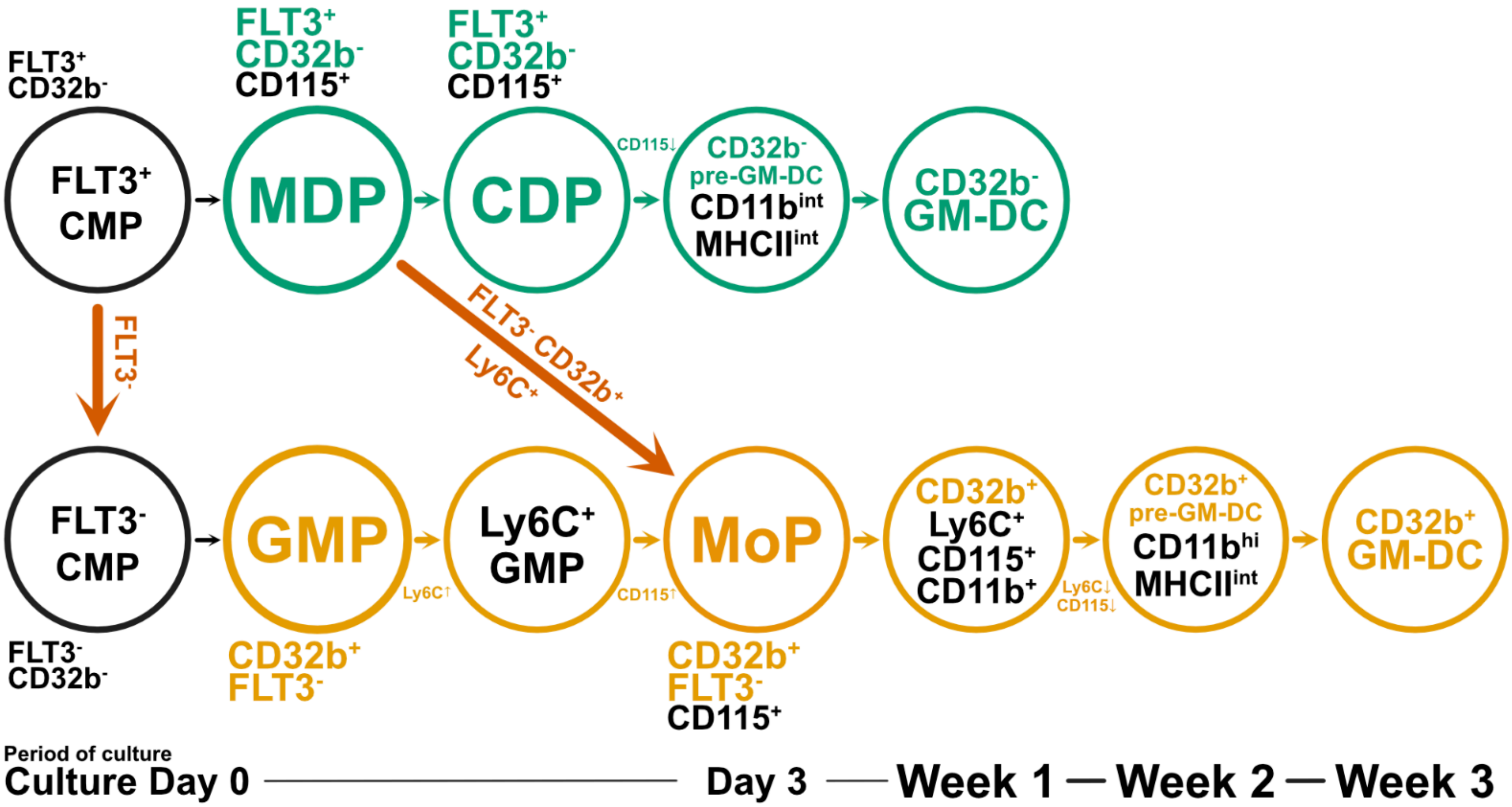

Supplementary Figure 18. Two-track model of GM-DC differentiation trajectories *in vitro*.

Schematic summary of the dual developmental pathways leading to GM-DC heterogeneity. The early differentiation track initiates from FLT3-expressing progenitors, including $FLT3^{+}$ CMPs, MDPs, and CDPs, which rapidly give rise to $CD32b^{-}$ pre-GM-DCs and $CD32b^{-}$ GM-DCs within the first week of culture. The late differentiation track arises from $FLT3^{-}$ CMPs, which serve as an intermediate population between $FLT3^{+}$ CMPs and GMPs. This track progresses through CD32b-sharing progenitors such as GMPs, $Ly6C^{+}$ GMPs, and MoPs. The pathway transitions through a $CD32b^{+}$ $Ly6C^{+}$ $CD115^{+}$ $CD11b^{+}$ intermediate state to form $CD32b^{+}$ pre-GM-DCs, resulting in the delayed emergence of $CD32b^{+}$ GM-DCs by Week 3.

SUPPLEMENTARY TABLE 1. Antibodies used in the study.

Antibodies	Source	Identifier
anti-mouse CD3 PE/Cy7 (clone 17A2)	Biolegend	Cat # 100219
anti-mouse CD3 APC (clone 145-2C11)	Biolegend	Cat # 100311
anti-mouse TCRβ chain PE/Cy7 (clone H57-597)	Biolegend	Cat # 109221
anti-mouse CD4 PE, PE/Cy7 (clone GK1.5)	Biolegend	Cat # 100408, 100421
anti-mouse Vα2 APC, PerCP/Cy5.5 (clone B20.1)	Biolegend	Cat # 127810, 127814
anti-mouse CD8a PE, PE/Cy7 (clone 53-6.7)	Biolegend	Cat # 100708
anti-mouse CD11b APC, PerCP/Cy5.5, BV421, BV605, PE/Cy7 (clone M1/70)	Biolegend	Cat # 101211, 101228, 101235, 101237, 101216
anti-mouse CD11c PE, BV421, APC/Cy7 (clone N418)	Biolegend	Cat # 117308, 117330, 117323
anti-mouse CD14 PE, PE/Cy7, FITC (clone Sa14-2)	Biolegend	Cat # 123309, 123316, 123307
anti-mouse CD16/32 PerCP/Cy5.5, PE (clone 93)	Biolegend	Cat # 101324
anti-mouse CD19 PE/Cy7, APC/Cy7 (clone 6D5)	Biolegend	Cat # 115520, 115529
anti-mouse CD24 PerCP/Cy5.5 (clone M1/69)	Biolegend	Cat # 101824
anti-mouse CD25 PerCP/Cy5.5 (clone PC61)	Biolegend	Cat # 102030
anti-mouse CD32b PE, APC (clone AT130-2)	eBioscience, Thermo Fisher Scientific	Cat # 12-0321-82, 17-0321-82
anti-mouse CD34 FITC (clone RAM34)	eBioscience, Thermo Fisher Scientific	Cat # 11-0341-82
anti-mouse CD40 PE (clone FGK45)	Biolegend	Cat # 157505
anti-mouse CD45 PE/Cy7 (clone 30-F11)	Biolegend	Cat # 103114
anti-mouse CD45.1 PE, BV421 (clone A20)	Biolegend	Cat # 110707, 110731
anti-mouse CD45.2 Alexa488, BV421 (clone 104)	Biolegend	Cat # 109816, 109831
anti-mouse B220/CD45R PE/Cy7 (clone RA3-6B2)	Biolegend	Cat # 103221
anti-mouse NK1.1 PE/Cy7 (clone PK136)	Biolegend	Cat # 108713
anti-mouse CD49b PE/Cy7 (clone DX5)	Biolegend	Cat # 108921
anti-mouse CD80 PE/Cy7 (clone 16-10A1)	Biolegend	Cat # 104733
anti-mouse CD83 APC (clone Michel-19)	Biolegend	Cat # 121509
anti-mouse CD86 PE/Cy7, PerCP/Cy5.5 (clone GL-1)	Biolegend	Cat # 105013, 105027
anti-mouse CD103 PE/Cy7 (clone 2E7)	Biolegend	Cat # 121425
anti-mouse CD115 Alexa647 (clone AFS98)	Biolegend	Cat # 347315
anti-mouse CD117 PE, Alexa647 (clone 2B8)	Biolegend	Cat # 105807, 105817
anti-mouse CD127 PE/Cy7 (clone A7R34)	Biolegend	Cat # 135013
anti-mouse FLT3 APC (clone A2F10)	Biolegend	Cat # 135310
anti-mouse CD172a FITC (clone P84)	Biolegend	Cat # 144006
anti-mouse DEC205 PE/Cy7 (clone NLDC-145)	Biolegend	Cat # 138209
anti-mouse CD206 PE/Cy7 (clone C068C2)	Biolegend	Cat # 141720

anti-mouse CD209a APC, Alexa488 (clone MMD3)	Invitrogen, Thermo Fisher Scientific	Cat # 50-2094-80, 53-2094-80
anti-mouse CD209b APC (clone 22D1)	Invitrogen, Thermo Fisher Scientific	Cat # 17-2093-80
anti-mouse CD301a PE/Cy7 (clone LOM-8.7)	Biolegend	Cat # 145609
anti-mouse CD301b PE/Cy7, APC (clone URA-1)	Biolegend	Cat # 146808, 146813
anti-mouse/rat XCR1 APC, PerCP/Cy5.5, FITC (clone ZET)	Biolegend	Cat # 148206, 148207, 148209
anti-mouse 33D1 PE, APC (clone 33D1)	Biolegend	Cat # 124905, 124913
anti-mouse Sca-1 APC/Cy7, PE/Cy7 (clone D7)	Biolegend	Cat # 108125, 108113
anti-mouse Ly-6C Alexa488, Alexa647 (clone HK1.4)	Biolegend	Cat # 128021, 128010
anti-mouse Ly-6G PE/Cy7, PerCP/Cy5.5 (clone 1A8)	Biolegend	Cat # 127618, 127616
anti-mouse F4/80 PE, PE/Cy7, PerCP/Cy5.5 (clone BM8)	Biolegend	Cat # 123109, 123127
anti-mouse H-2 PE (clone M1/42)	Biolegend	Cat # 125506
anti-mouse I-A/I-E APC/Cy7, Alexa488 (clone M5/114.15.2)	Biolegend	Cat # 107628, 107616
anti-mouse PD-L1 PE/Cy7 (clone 10F.9G2)	Biolegend	Cat #124313
anti-mouse PD-L2 PE/Cy7 (clone TY25)	Biolegend	Cat # 107213
anti-mouse CLEC12A APC (clone 5D3/CLEC12A)	Biolegend	Cat # 143405
anti-mouse CX3CR1 Alexa647 (clone SA011F11)	Biolegend	Cat # 149003
anti-mouse CCR6 PerCP/Cy5.5 (clone 29-2L17)	Biolegend	Cat # 129809
anti-mouse CCR7 PE (clone 4B12)	Biolegend	Cat # 120105
anti-mouse TER119 PE/Cy7 (clone TER-119)	Biolegend	Cat # 116221
Rat IgG1, κ isotype Ctrl PE, PE/Cy7, APC, BV421, APC/Cy7, PerCP/Cy5.5 (clone RTK2071)	Biolegend	Cat # 400407, 400416, 400411, 400429, 400422, 400425
anti-mouse CD115 biotin (clone AFS98)	Biolegend	Cat # 135508
anti-mouse CD135 biotin (clone A2F10)	Biolegend	Cat # 135308
anti-mouse CD11b biotin (clone M1/70)	Biolegend	Cat # 101204
anti-mouse CD19 biotin (clone 6D5)	Biolegend	Cat # 115504
anti-mouse CD25 biotin (clone PC61)	Biolegend	Cat # 102004
anti-mouse CD44 biotin (clone IM7)	Biolegend	Cat # 103004
anti-mouse NK1.1 biotin (clone PK136)	Biolegend	Cat # 108704
anti-mouse I-A/I-E biotin (clone M5/114.15.2)	Biolegend	Cat # 107604
anti-mouse F4/80 biotin (clone BM8)	Biolegend	Cat # 123106
anti-mouse CD4 biotin (clone GK1.5)	Biolegend	Cat # 100404
anti-mouse CD8 biotin (clone 53-6.7)	Biolegend	Cat # 100704
Streptavidin PE, Alexa488	Biolegend	Cat # 405245, 405235
